# Form for planning and elaborating high fidelity simulation scenarios: A validation study

**DOI:** 10.1371/journal.pone.0274239

**Published:** 2022-09-28

**Authors:** Regina Mayumi Utiyama Kaneko, Inês Monteiro, Maria Helena Baena de Moraes Lopes

**Affiliations:** 1 Treinamento Desenvolvimento & Educação–SIMSAFETY, São Paulo, São Paulo, Brazil; 2 Faculty of Nursing, University of Campinas, Campinas, São Paulo, Brazil; Bilawal Medical College, Liaquat University of Medical and Health Sciences, PAKISTAN

## Abstract

Every human being has the right to safe, dignified and harm-free care in health institutions. High fidelity simulation has been used in teaching for the training and continuing education of health professionals to promote quality, safe and humanized patient care. Elaborating scenarios is an important phase to provide a simulation-based experience, and is relevant in the teaching-learning process. The objective of this study was to validate the content and applicability of the High Fidelity Simulation Scenario Planning and Development Form and its Operational Manual. The form could be used to development of scenarios to medicine, nursing, physiotherapy and as well as other specialties in the healthcare. This was a methodological validation study of the form and its manual content by experts in simulation and its feasibility, conducted in two phases: Phase 1: eight experts were selected using the “snowball” sampling technique to validate the content measured by the content validity index; Phase 2 (test): the form and its operational manual validated by the experts were made available to 28 participants in order to elaborate scenarios for the feasibility assessment and participation in the focus group. All items in the form and in the operational manual reached a content validity index above 0.80. The total content validity index was 0.98. The evaluation of the usability of the instruments carried out by the participants reached a percentage above 96.43% in all alternatives except for the item “It was easy to use the form to build your scenario” (75%). Eight participants were present in the focus group. Focus group discussions were categorized into completeness, practicality and usefulness according to comments and suggestions. The form and its operational manual proved to be valid instruments.

## Introduction

Safe care at different healthcare levels is a social, professional and ethical duty to mitigate the alarming numbers of deaths caused by adverse events during procedures in healthcare institutions [1, 2]. At least 50% of these events are preventable with preventive actions supported by health policies [3, 4]. Therefore, inserting simulations with the use of health scenarios into the curriculum and selecting this strategy in continuing education programs is necessary to contribute and add to the education and training of students and professionals with the purpose of providing safe and quality care [5–7], and necessary for strengthening the organizational culture in terms of patient safety [8, 9].

Studies demonstrate the importance of using evidence-based interactive and reflective teaching and learning strategies in training health professionals [10, 11], and one of the strategies is healthcare simulations, as they provide experience based on real cases in a controlled and interactive environment to improve technical and non-technical skills [12–14]. Healthcare simulation is a methodology, not exclusively a technology, that has been increasingly used to amplify real experiences with guided experiences that evoke or replicate substantial aspects of the real world, being very well highlighted as a technique and not a technology [15]. This clinical scenario experienced by the participants and subsequent discussion in the debriefing to re-examine the correct actions and opportunities for improvement identified by the participants stimulate critical and reflective thinking to consider maintaining good practices or changing them [16–18], as well as collaborative and interprofessional practices [19, 20] to develop and maintain effective behaviors and technical skills.

Essential elements for healthcare simulation structure are recommended by the Best Evidence in Medical Education (BEME) [21], International Nursing Association for Clinical Simulation Learning (INACSL) [22–25] and the International Association for Medical Education in Europe (AMEE) [26] as good practices in simulation, and demonstrate direct implication in the planning and elaboration of scenarios [27], as well as in training professionals involved with this practice.

The growth of simulation societies has contributed to the dissemination of good practices for their application, contributing to a safe practice, and the improvement of the quality of health care practice. In addition, advances in research and innovations are shared with professionals around the world. Among them, the Society for Simulation in Healthcare, Society for Simulation in Europe and others that stand out as great disseminators of the strategy.

Despite the growing use of the health simulation technique in teaching and healthcare institutions based on theoretical references [21–26], there is a scarcity of instruments in the literature for elaborating healthcare simulation scenarios which have an operational manual with guidelines and that are validated by experts and the target audience in terms of feasibility assessment [28].

The Healthcare Simulation Scenario Planning and Elaboration Form (*Formulário de Planejamento e Elaboração de Cenário de Simulação Realística em Saúde ForPEC*) was developed by the lead author of this article in 2015 and has been continuously improved according to its use in elaborating scenarios and its application in developing technical and non-technical skills of Healthcare professionals. Therefore, our objective was to validate the content and feasibility of the Healthcare Simulation Scenario Planning and Development Form and its Operational Manual.

## Method

### Study design

This is a methodological content validation and feasibility assessment study. To evaluate content of forms the determination of experts is a crucial step for this evaluation. After determining the experts, the items and entire forms as content valid have to be established. The Index of Content Validity (CVI), which is derived from a rating scale, where one connotes an irrelevant item and four an extremely relevant item was used to evaluate the content of forms. The CVI is the proportion of items that received a rating of three or four by the experts [29, 30].

#### Study location

The experts who evaluated the content were affiliated with public and private hospitals, educational institutions, simulation centers, and skills laboratories in public and private institutions in the cities of São Paulo, SP, Campinas, SP, Presidente Prudente, SP, and Natal, RN, Brazil.

The facilitators who participated in the test and assessed the feasibility of the instrument and its manual work in the city of São Paulo, SP, Brazil.

#### Population and sample

The study was developed in two phases. First, a group of experts assessed the content validity of the *ForPEC*, the operational manual and the feasibility assessment questionnaire. A test was subsequently conducted with professionals who work in simulation (facilitators) to elaborate a scenario using *ForPEC* and its already validated operational manual. Then, eight experts were invited [30] and the “snowball” sampling technique was performed [31]. The first invited professional was asked to nominate others. It was established that there would be 30 facilitators for the test [32] and the “snowball” sampling technique was performed by convenience [30].

#### Inclusion, exclusion, and discontinuation criteria

The Lattes Platform was consulted to select experts, and the attributes of the expert concept proposed by Jasper [33] were also adopted.

The Lattes Platform [34] represents CNPq’s (The National Council for Scientific and Technological Development) experience in the integration of Curriculum, Research Group and Institution databases. Its current dimension extends not only to the actions of planning, management and operationalization of CNPq’s funding, but also of other federal and state funding agencies, state foundations to support science and technology, higher education institutions and institutes of search. In addition, it has become strategic not only for planning and management activities, but also for the formulation of policies by the Ministry of Science and Technology and other government bodies in the area of science, technology and innovation.

As the attributes are broad, some specific criteria were defined for the present study and professionals who met at least one criterion for each attribute were selected as experts. The attributes and their respective criteria are described below.

*Attribute: Possession of a specialized body of knowledge or skill*.

Criterion 1: participates in training and updating courses related to healthcare simulation;
Criterion 2: regularly participates in national and/or international congresses on healthcare simulation (at least once a year);
Criterion 3: is a member of a healthcare simulation association and/or society.

*Attribute: Extensive experience in that field of practice*.

Criterion 1: develops training or qualification programs or healthcare simulation assessments;
Criterion 2: develops programs of academic activities in healthcare simulation;
Criterion 3: elaborates technical and/or behavioral scenarios;
Criterion 4: has professional experience in healthcare simulation;
Criterion 5: is a facilitator of technical/behavioral scenarios in healthcare simulation.

*Attribute: Highly developed levels of pattern recognition*.

Criterion 1: trains professionals and/or facilitators in healthcare simulation;
Criterion 2: instructs professionals working in skills labs and simulation centers;
Criterion 3: holds a Master’s and a Doctorate degree in a line of research related to healthcare simulation.

*Attribute: Acknowledgement by others*.

Criterion 1: is or was manager of skill labs and healthcare simulation centers;
Criterion 2: is the author of scientific articles, books or book chapters on the topic of healthcare simulation;
Criterion 3: gives lectures, conferences and courses at scientific healthcare simulation events;
Criterion 4: participates or has participated on the board of directors of companies and associations related to healthcare simulation;
Criterion 5: participates in an evaluation panel of works in post-graduation *Stricto sensu* in research lines involving simulation.

The experts evaluated the *ForPEC* items, its operational manual and the feasibility questionnaire in this first stage regarding the relevance/representativeness, clarity, comprehensiveness and general appearance of the instruments.

The expert committee was composed of: 1) two registered nurses; 2) two doctors; 3) two professionals from the multidisciplinary team (speech therapist and biomedical scientist); and 4) two professors.

Those who had completed at least one training course in simulation and/or had been facilitators and/or had constructed at least 10 technical or behavioral scenarios in the health area were considered eligible for the test.

Participants who: withdrew their consent to the study, did not meet the established deadlines, or did not complete parts III (Learning in Results), V (Logistics of the simulation center) and VI (Full description of the case) of the form were expected to be discontinued. of planning or did not elaborate the scenario.

#### ForPEC and its operational manual

The Healthcare Simulation Scenario Planning and Elaboration Form (i.e. *Formulário de Planejamento e Elaboração de Cenário em Health Care Simulation*–ForPEC) version 1.1, was based on best practices in simulation [21–26] and contains 10 sections in a total of 91 sub-items: 1) general planning; 2) minimum requirements; 3) learning and results; 4) references for elaborating the content and submitting the material for pre- or post-simulation reading; 5) simulation center logistics; 6) full description of the case; 7) laboratory and imaging test results; 8) debriefing; 9) checklist and 10) case reading (S1 File).

The Operational Manual version 1.0 is a tool with instructions to guide completing the *ForPEC*. It contains 43 pages and the 91 *ForPEC* items are presented with their respective descriptions and guidelines to guide the scenario elaboration. It presents two example scenarios: technical scenario (chest tube) and non-technical scenario (communicating bad news) (S2 File).

#### Feasibility assessment questionnaire

This questionnaire was developed specifically for this study and contains six questions, with five (three related to ease of filling and two to completeness) being presented as statements. The agreement was evaluated using a Likert-type scale that ranged from 1 (totally disagree) to 5 (I totally agree). An open question evaluated whether any item was missing on the *ForPEC* or in the operational manual, with “yes” and “no” answer options, in addition to an open field to indicate the items that were missing.

#### Content validation

The Content Validity Index (CVI) of each instrument item and the total CVI was calculated [29, 30] to verify the content validity of the form, manual and feasibility assessment questionnaire. The CVI was calculated through the total number of “3” or “4” responses given by the experts to a given item and dividing it by the total number of expert responses, as presented in the following formula [29, 30]:


CVI=Totalnumberof“3”or“4”responsesfromexperts/totalnumberofexpertsresponses.

The pertinence/representativeness, clarity, scope and general appearance of the instruments were evaluated. A four-point Likert scale to evaluate the pertinence/representativeness, clarity, scope and general appearance if the instruments was used for assessment.

The maximum value of the CVI is 1.00. A CVI equal to or greater than 0.80 was considered adequate [29, 30]. Items that did not reach the mimium value of 0.80 would be reviewed and sent to the experts for a new validation process [29, 30].

#### Test

Facilitators developed scenarios and analyzed the feasibility of *ForPEC* and its operational manual. This step involved planning and elaborating a technical or behavioral scenario, carrying out the pilot/testing of the built scenario and individually and independently evaluating the feasibility of *ForPEC* and its Operational Manual. The scenario theme was free to choose.

After elaborating the scenarios and carrying out the pilot/test, all test participants were invited to participate in the focus group [35]. The objective was to discuss the perception and experience in relation to the elaboration process and conduction of the pilot/test of the scenario with the use of these resources. Two focus groups with at least six participants were established [35].

The audio and video recordings of the discussions were made in a MOV file and fully transcribed in the Microsoft Office Excel^®^ spreadsheet editor. The audio and video recordings made in the face-to-face activity of the focus group were transcribed in full and grouped according to the *ForPEC* parts into three categories: completeness, practicality and usefulness.

#### Ethical aspects

The data collected during the study were anonymously documented and the experts and participants were only identified by a number and their initials. The process began only after the study was approved by the Research Ethics Committee (CEP) of the State University of Campinas (UNICAMP) and signing the free and Informed Consent Form (ICF). The project was submitted and approved by the Research Ethics Committee of the University of Campinas, UNICAMP under the opinion no. 2.302.587, 28/09/2017, CAAE: 72812217.0.0000.5404.

Participants were included in the study, only after obtaining the written consent, signed and dated in two copies. One copy was kept by the participant, and the other was collected by the study author. The informed consent form and all study documentation (Form, manual, explanatory letter) were delivered personally and/or by mail by the researcher (the fisrt author).

## Results

Nine professionals were invited and eight agreed to participate as experts. All had five or more years of experience in simulation and worked in more than one area (teaching, research, assistance or management). (Table 1) It was not necessary to conduct individual interviews.

**Table 1:** Distribution of experts according to time of experience in healthcare simulation and field of work (n = 8), Brazil, 2018.

Experts	Time of experience in healthcare simulation (years)	Field of work
Registered Nurse	6,25	Teaching/Research/Management
Registered Nurse	11,25	Teaching/Research/Management
Registered Nurse	10,58	Teaching/Research/Management
Registered Nurse	5	Teaching/Research/Management
Biomedical Scientist	10	Teaching/Research/Management
Speech therapist	8,42	Teaching / Management
Physician	22	Teaching / Research /Practitioner
Physician	8	Teaching / Research /Practitioner

Physicians, in addition to their roles in teaching and research, were practitioner, one as an emergency physician and the other as an emergency physician and pediatrician.

The experts met 81.25% to 100% of the criteria for each attribute. The expert who presented the lowest percentage (68.75%) met the lowest number of criteria for the attribute “recognized as authority” (Table 2).

**Table 2:** Selection of experts according to the attributes proposed by Jasper [33] and modified for this study (n = 8). Brazil, 2018.

Attributes	Criteria	n	%
Possession of a specialized body of knowledge or skill	Instructs professionals working in skill labs and healthcare simulation centers	8	100.00
	Regularly participates in national and/or international congresses on healthcare simulation (at least once a year)	8	100.00
	Member of a healthcare simulation association and/or society	8	100.00
Extensive experience in that field of practice	Develops training programs or qualifications or evaluations with the healthcare simulation strategy	7	87.50
	Develops academic activity programs with the healthcare simulation strategy	8	100.00
	Develops technical and/or behavioral scenarios	8	100.00
	Has professional experience in the healthcare simulation strategy	8	100.00
	Facilitator of technical and/or behavioral healthcare simulation scenarios	8	100.00
Highly developed levels of pattern recognition	Trains professionals and/or facilitators in the healthcare simulation methodology	8	100.00
	Instructs professionals working in skills labs and simulation centers	8	100.00
	Holds a Master’s and Doctorate degree in a research line related to healthcare simulation	6	75.00
Acknowledgement by others	Is or was manager of skills labs and simulation centers with the healthcare simulation strategy implemented	8	100.00
	Is the author of scientific articles, books or book chapters with the theme healthcare simulation	7	87.50
	Gives lectures, conferences and courses at scientific healthcare simulation events	7	87.50
	Participates or has participated in the board of companies and associations related to healthcare simulation	5	62.50
	Participates in a review board of works in postgraduate *Stricto sensu* research lines involving simulation	5	62.50

All of the *ForPEC*—version 1.1 and the operational manual—version 1.0 items reached CVI above 0.80, except for items 83—Evolution of *ForPEC* and item 10—Level of the operational manual, which presented CVI of 0.75 in relation to clarity. These two items were then modified according to the experts’ suggestions, and sent for reassessment (*ForPEC*–version 1.2 and Operational Manual–version 1.1). In the end, both reached a CVI of 1.0. After the second round, the total CVI of the *ForPEC*–version 1.2 was 0.98 and that of the Operational Manual–version 1.1 was 0.98, as shown in Table 3.

**Table 3:** Expert agreement on the scope of each part of the *ForPEC* and the Operational Manual (n = 8). Brazil, 2018.

Items	Final CVI (*ForPEC*)	Final CVI (Operational Manual)
Part I: General planning	1.00	1.00
Part II: Minimum Requirements	1.00	1.00
Part III: Learning outcomes	0.88	0.88
Part IV: References for content preparation and submission of material for pre- or post-simulation reading	1.00	1.00
Part V: Simulation Center Logistics		
Volunteer information	1.00	1.00
Duration	1.00	1.00
Technological and Human Resource to compose the character of the scenario	1.00	1.00
General orientations	0.88	0.88
Monitoring	1.00	1.00
Accesses	1.00	1.00
Equipment and Materials	1.00	1.00
Environment	1.00	1.00
Makeup/Moulage and Accessories	1.00	1.00
Part VI: Full description of the case		
Scenario	0.88	0.88
History and Character Anamnesis	1.00	1.00
Technical information	1.00	1.00
Character Profile Description	1.00	1.00
Scenario evolution	1.00	1.00
Part VII: Exam Result	1.00	1.00
Laboratories or Reports	1.00	1.00
Images	1.00	1.00
Part VIII: Debriefing	1.00	1.00
Part IX: Competency Performance Checklist/Analysis	0.88	0.88
Part X: Reading the Case to the Participant	1.00	1.00

Despite the other items of *ForPEC*–version 1.2 and the Operational Manual–version 1.1 receiving CVI ≥ 0.80, they received 108 suggestions and comments. Thus, 79 of them were incorporated into the *ForPeC* and the Operational Manual, producing the final versions: *ForPEC*–version 1.3 and the Operational Manual–version 1.2.

The experts evaluated the layout of *ForPEC* and the Operational Manual with a CVI of 1.0. An expert commented that the form was extensive and advised checking the possibility of synthesizing.

A feasibility assessment questionnaire for *ForPEC* and its Operational Manual were developed for implementation in the second stage of the study, which was evaluated by experts. The CVI received a score of 1.0 in terms of relevance, clarity and comprehensiveness in all items.

A total of 32 health professionals were invited and 30 agreed to participate in the test, signing the informed consent form. (Table 4) All of them built the scenario and answered the feasibility assessment questionnaire of the *ForPEC* version 1.3 and its Operational Manual version 1.2. Two participants were later excluded for not completing part VI (Full Description of the Case). Among the 28 professionals who remained in the study, eight (seven nurses and one doctor) divided into groups of four members only participated in the focus group once. Most facilitators were nurses (85.72%) and working in the teaching area (96.43%).

**Table 4:** Demographic of health professionals—test (n = 28). São Paulo, 2018.

	n	%
**Gender**		
Male	5	17,86
Female	23	82,14
**Age group (years)**		
26 to 36	21	75,00
37 to 47	5	17,86
48 to 58	2	7,14
**Type of organization**		
Public	10	35,71
Private	16	57,14
Both	2	7,14
**Academic education**		
Registered Nurse	18	64,29
Registered Nurse and Simulation Technician	6	21,43
Physician	4	14,29
**Field of work**		
Teaching	20	71,43
Management	1	3,57
Teaching/Practitioner	1	3,57
Teaching/Research/Practitioner	6	21,43

It is observed in Table 5 that the participants were experienced in building scenarios, having elaborated from 5 to 59 scenarios or more. It is noteworthy that one participant reported having prepared and debriefed 405 scenarios.

**Table 5:** Distribution of participants according to professional experience in the use of the healthcare simulation technique (n = 28). Sao Paulo, SP, Brazil 2018.

Variables	n	%
Experience time (years)		
1 to 2	10	35.71
3 to 4	9	32.14
5 to 6	6	21.43
7 to 8	3	10.71
Completed courses (number)		
None	3	10.71
1 to 3	14	50.00
4 to 6	10	37.72
7 or more	1	3.75
Scenarios built (number)		
5 to 15	17	60.71
16 to 26	5	17.86
27 to 37	3	10.71
38 to 58	1	3.57
59 or more	2	7.14
Scenario facilitator (number of times)		
None	3	10.71
1 to 20	15	53.57
21 to 41	2	7.14
42 to 62	4	14.29
≥ 63	4	14.29

All test participants completed the feasibility assessment questionnaire after the scenario was developed. Statements 1, 2 and 5 reached 100% agreement, and statement 4 96.43% (strongly agree and agree). In relation to statement 3 (It was easy to use the *ForPEC* form to build your scenario), it was observed that 25% indicated I do not agree and disagree (Table 6).

**Table 6:** Feasibility assessment of the *ForPEC* and its Operational Manual by participants after scenario development (n = 28). Sao Paulo, SP, Brazil, 2018.

Items	1. The *ForPEC* Form covered all the items for building your scenario.		2. The Operational Manual for completing the *ForPEC* included all the instructions for building your scenario.		3. It was easy to use the *ForPEC* form to build your scenario.		4. The Operational Manual was useful for building the scenario in *ForPEC*.		5. The examples of scenarios present in this manual facilitated understanding and construction of the scenario.	
	n	%	n	%	n	%	n	%	n*	%*
Completely agree	17	60.71	16	57.14	11	39.29	15	53.57	19	70.37
Agree	11	39.29	12	42.86	10	35.71	12	42.86	8	29.63
Do not agree or disagree	0	0.00	0	0.00	4	14.29	1	3.57	0	0.00
Disagree	0	0.00	0	0.00	3	10.71	0	0.00	0	0.00
Completely disagree	0	0.00	0	0.00	0	0.00	0	0.00	0	0.00

*n = 27 (a user did not respond because he did not use the examples to build the scenario).

Regarding item 6 of the feasibility questionnaire “Are there any items missing from the *ForPEC* or the Operational Manual?”, 14% answered yes. They suggested adding items to guide actors through the “turning point” of their lines, meaning when the actor must change their performance according to the reaction of the volunteer participants in the scenario. Other suggestions were: informing the patient’s age, increasing the space for recording the phrases of expected conduct in item 80 (Phrases that could be used) and description of the special conditions of the scenario, for example, relevant information about the way of working and availability of resources.

Most of the scenarios developed by the participants (75%) were aimed at developing technical skills, as shown in Table 7.

**Table 7:** Titles of the scenarios developed by the participants (n = 28). Sao Paulo, SP, Brazil, 2018.

Scenario titles	N	%
*Technical scenarios*	21	75
Child admission to the emergency room		
Identification and treatment of Cardiopulmonary Arrest—Pre-Hospital Care		
Anticholinergic Syndrome		
Acute myocardial infarction		
Basic life support		
Adult Cardiopulmonary Arrest		
“I ventilate but do not intubate”		
Hemorrhagic code triggering		
Medication administration		
Respiratory failure in the newborn		
Cardiac arrest care		
Cardiological emergencies and emergencies		
Patient care maintaining chest tube		
Stroke identification		
Pulmonary edema		
Septic shock		
Septic shock in pediatrics		
In-hospital cardiac arrest		
Emergency care—arrhythmias		
Febrile convulsion		
*Behavioral scenarios*	**5**	**17.86**
Assertive communication		
Treatment adherence		
Routine consultation—violence against children		
Cognitive bias—cognitive error		
Communicating bad news		
*Technical and behavioral scenarios*	**2**	**7.14**
Fall precaution guidance for patients with high expectation		
Conflict management		

The focus group were held at the Simulation Center with infrastructure and resources for audio and video recording. Free speech was respected, with minimal intervention when the group distanced itself from the theme of the meeting. The first focus group lasted 48 minutes and the second 74 minutes.

The test participants chose one of the two dates available for the focus group. The footage was transcribed in full, analyzed and categorized as to completeness (15 comments), practicality (nine comments) and usefulness (four comments), according to textual statements identified by the number assigned to each professional, as presented below:

Completeness

*P15 In general*, *it helps in elaborating scenarios and especially for those who have no experience*, *it reminds them of the necessary items*, *I felt this ease*. *For example*, *making it clear to the facilitator*, *list the materials such as*: *probe*, *catheter*, *alcohol gel*, *bracelet; because when the person is starting to make a scenario*, *we can forget about it*.

*P15 For example*, *mine was a bigger set and lacked space*. *Overall*, *I really enjoyed the job*. *It will help a lot*.

*P15 There was no space for some things and there are spaces that I didn’t use*. *Overall*, *I found it very good*

*P15 Nothing was missing*

*P5 I felt a lack of space to write*

*P5 It caught my attention*, *initially*, *when I didn’t fill in all the items*. *I thought if I should only focus on the technical or only on the behavioral*, *but as I progressed in the construction*, *I realized that they are together*. *This was clear because I made a technical scenario and I soon realized that I can use a single form to also create a behavioral scenario and/or integrate these skills*

*P15 I wouldn’t exclude anything*. *There were fields that I didn’t use and it’s understandable that it’s not possible to delete them*. *Because it is a very didactic instrument for people to remember things depending on the scenario*. *There were fields that I didn’t really use*, *but my scenario wasn’t like the others*, *which were more technical*

*P5 I had no idea that for creating the scenario we do all that*, *92 items*! *This transformation into something tangible was interesting*. *There are 92 items to make a suitable scenario*, *reach the objective*, *discuss the objectives*. *Somehow what was just in our mind became very real and with direction*

*P3 The patient didn’t speak in my scenario*, *but there is no way to exclude it*, *you direct something*

*P2 The form is complete*, *my scenario is from Basic Life Support and some fields were not filled in*, *such as imaging*, *laboratory tests*. *However*, *having a complete form is very good because we meet internal and external demands where I work*. *They contact us and we produce the scenario*. *Contact is often on the day of training*, *for example*, *if it will progress to a cricothyroidotomy*. *When we have this information described in advance*, *I believe it will help many simulation centers and skill labs for advance planning*.

*P1 I developed a pediatric trauma care scenario*. *It was an internal demand for training at the hospital*. *I was having a little fun with the instrument*. *I put the data according to the age group and following the step by step so I don’t get lost or missing elements or even insert items that I wouldn’t use in the scenario*. *It was very practical*.

*P29 Nothing was missing from the form*

*P6 I thought creating scenerios is really hard*, *congratulations*! *Because how many things do we need to see to set up a scenario and there is a need for this detail when we work with a technician or intern*, *but it seems to me that there is repeated information*

*P7 The form helped a lot in terms of selecting all the necessary items without running the risk of forgetting any point*, *you are forced to go through the steps and think*, *the script has this very strong role*. *Sometimes you turn on autopilot and forget items for scenario building*. *It was pertinent to enter everything in the form and you determine what will be needed in your scenario*. *So*, *I thought it was very good*, *thought of every detail*. *I really enjoyed it*

*P29 TUPASS has open spaces and you forget to write everything*. *This manual and form include all the details*

Practicality

*P15 I’ve been using simulation for some time and the form helped in terms of scenario planning*, *it’s easy*. *I answered the feasibility assessment questionnaire and for the most part I completely agreed with everything*. *There was one that I only agreed with*, *in the question*: *It was easy to use the ForPEC form to build your scenario*, *because I thought it lacked space to write*

*P15 I developed guidelines for precaution against falls in patients with high expectations of care*, *which is our target clientele where I work*. *I decided to create the scenario in the form for us to work on our fall indicator*. *There is a form that the patient needs to sign*, *but it is not associated with guidelines for falling*. *You approach the patient and ask*, *were you advised about the fall*? *The patient answers*: *no*. *But didn’t the nurse tell you that you can’t get out of bed by yourself*? *Yes*, *she did*. *Didn’t the nurse say to put sneakers on to walk in the hallway*? *Yes*, *she did*. *So*, *this is all fall guidelines*. *It is a very didactic tool for people to remember things depending on the scenario and I managed to put all of that together*.

*P15 I thought it took some time because it has a lot of detail*. *It’s good because nothing goes unnoticed*. *I didn’t measure time*.

*P3 I found it a little time consuming in the elaboration and I was in the pilot test*. *We used a shorter one in our service and every time we ran the scenario*, *we had to go back to add things*. *With this form*, *when we performed the test*, *nothing was missing and we even gained time without the need for adjustments*

*P5 You spend more time on designing and less time on redoing*. *I still thought I would do a Basic Life Support and I didn’t imagine it would take so long*! *When I started the elaboration*, *I realized the amount of items needed*, *it’s wonderful*!! *And so*, *you don’t make mistakes*, *right*?

*P6 I thought it was very good*, *because there are things we do automatically and we don’t think*

*P29 I didn’t look at the manual before*, *I tried to fill it in and found it intuitive*. *I barely used the manual*

*P6 It’s practical and forces you to think*

*P7 It’s very objective and will be very useful*

Utility

*P2 It will help professionals who start in the area and don*’*t yet have the expertise with the method*

*P2 It was very important*, *including to discuss our workflows internally*. *Currently*, *the professional sketches the idea*. *With the form they will be able to fill in the information and we optimize the time of everyone involved*

*P15 For the simulation center*, *which is my reality*, *the logistics part*, *for example*, *the simulation operation*, *you leave it to the simulation center; for the other items*, *the professional who will use the simulation center will reserve a day to determine the items required in advance*: *duration*, *type of simulator and makeup*. *This will help*!

*P2 My reality would be a partnership*, *for example*, *the logistics part is with the simulation center*. *They can set aside a day for discussion and send what they will need in advance*: *time*, *low-fidelity simulator*, *makeup*, *etc*. *I would not ask you to fill in the other parts of the form*. *“Elaborating the scenario on the form is the right thing to do for any professional*. *It’s horrible when the professional doesn’t have the conscience and when asked to elaborate the case*, *they answer*: *it’s not necessary*, *we’ll change the parameters at the time of training*. *It’s bad*.*”*

## Discussion

There is a scarcity of instruments for elaborating healthcare simulation scenarios and an operational manual with guidelines for completion validated by experts in the literature, despite the increasing use of this technique in educational and health institutions based on theoretical references [21–26].

Some models [36] have conceptual elements for development, but they are not oriented for implementing scenarios. Others direct the construction of scenarios aiming at developing technical skills, but there are limitations for the non-technical competence because they do not provide guidelines for the actors regarding the profile of the characters, lines and others [37]. There are also models that have gaps in terms of logistics (materials, equipment, audiovisual resources and others), the scenario purpose, strategies for monitoring indicators and expected results for the institution [38, 39].

A recent study provides a tool for evaluation of written simulation scenarios validated. They presented essential elements for this evaluation: learning objectives, clinical context/scenario overview, critical actions, patient states, scenarios materials and resource [40]. The use of tools favors the development of scenarios and consequently the success of a simulation. Additionally, they are important processes for the implementation of good practices associated with other tools that allow the evaluation of the scenario writing. *ForPEC*, in addition to the essential elements for evaluation of written simulation scenarios, there is the possibility of writing the general planning, logistics of the scenario, characterization of the standard patient, important elements for conducting the debriefing and checklist.

Submitting *ForPEC* to the validation process by experts and evaluating its feasibility by the target audience was useful to provide a valid and practicable instrument to professionals who use this methodology in their activities. Additionally, the Operational Manual, developed and submitted for validation by experts, was important for the user to correctly fill in the instrument and be able to customize it to their reality.

The use of attributes suggested by Jasper [33] and the criteria that were adapted for this study favored selecting specialist experts and ratified the need for this choice to be based on knowledge, high-level skills, performance and intensive experience with simulation, constituting elements which make them experts recognized by their peers. Fifty percent of the experts fully met the attribute “recognized as authority” and the rest partially. This is due to the fact that it requires a high level of knowledge and experience. The attributes proposed by Jasper [33] were useful for the choice of specialists, with the attribute “recognized as authority” having greater weight [41, 42]. In fact, experts who met this attribute contributed to improving ForPEC and its Manual.

The content validity result of *ForPEC* and its Operational Manual reached a total CVI of 0.98; however, the suggestions and comments made by the experts in returning the material sent were of great relevance and magnitude, allowing to incorporate improvements into *ForPEC* and its Operating Manual.

The high number of suggestions and comments presented by the experts were relevant, and 73% were incorporated into both the *ForPEC* and its manual, evidencing the importance of the experts’ contribution to improve the material produced. As examples, we can mention the suggestions of: inserting the real photo of the wound with necrosis or lesion with excoriation in the form, in addition to the description to help perform the makeup/moulage [43]; establishing the duration of selected sounds and their interruption (i.e. child’s cry or noise and siren) in a way that is coherent with reality [44, 45]. It was suggested to remove the full name, medical record number, bed number and date of birth; however, these data were kept in the *ForPEC*, since the scenarios need them to make an identification bracelet, check the patient’s medical record [46], identify the blood bag in blood transfusions [47], tubes for collecting laboratory tests and serums. In fact, these findings reinforce the importance of including seemingly irrelevant items, but in the context of scenario development, it can generate a series of learning opportunities, such as patient identification, which is an international goal of patient´s safety. These are relevant elements for realism, scenario fidelity and patient safety with an impact on the development of clinical skills.

The use of instruments for scenario development favors applying high-fidelity simulation [21–26, 48] and provides synergy with care practice in terms of patient safety [49–52]. For example, carrying out the pilot/test of the scenario prepared by the participants in the test is considered as an element of good practices [22], and identified opportunities for improving the scenario execution and pointed out latent threats which favored reflection and implementation behavior in professional practice. Studies demonstrate the importance of the pilot for inserting high-fidelity simulation in the curriculum and in implementing them in hospital units [53, 54].

The feasibility assessment of the instruments reached a percentage above 96.43% in almost all items. Although the facilitators considered the complete instruments and the examples of scenarios provided in the operational manual to facilitate scenario construction, the ease of use did not reach the same agreement percentages. Participants reported data repetition in part V (Simulation Center Logistics) and VI (Full Case Description). The need for spaces to create the clinical evolution triggers in the simulator was also reported as an opportunity for improvement to facilitate scenario conduct.

The use of the focus group made it possible to capture valuable impressions of the quantitative assessment data of the instruments and generated responses from the discussions. The scenarios constructed by the professionals covered several multidisciplinary topics, with an increased interdisciplinary educational approach in clinical simulations [55, 56]. One of the scenarios built addresses violence against children and these are situations in which the interdisciplinary approach can be carried out by health professionals from identifying signs of abuse, reception, knowledge of the legislation and empathy.

Regarding the focus group discussions, *ForPEC* was perceived as a complete, flexible and adapted guideline for the specific construction of a scenario in the completeness category, which also addresses non-technical skills, even in scenarios with technical objectives.

*ForPEC* was also considered easy, practical, intuitive and directing, although the facilitators used a longer time in the creation due to the need for detailing, it was productive in conducting the pilot because there was only the need for small adjustments.

The utility of the *ForPEC* was perceived as an aid to professionals involved with the technique, especially for beginners. It was reported how the instruments helped them in the logistics, in establishing objectives and in inserting resources (for example, use of mat and walker in the scenario about risk of falling) in elaborating the scenarios. The *ForPEC* helped to list all the materials needed. Then, they validated where each resource would be to start the scenario in the pilot/test of the scenario, such as the mat next to the bed and the walker away from the bed to develop reasoning about the risks of falling during the scenario.

Although some training centers divide the team of facilitators to perform certain tasks such as scenario construction, simulator operation, debriefing and others, it is essential that everyone experiences the debriefing, as this step is considered essential for simulation based on good practices [22]. Studies corroborate the importance of debriefing for developing skills and behaviors in the teaching-learning process [57–59].

Just as guidelines and protocols are living documents and must be updated according to research and practical application, guidelines and models can and should be modified. It is desirable that these tools guide scenario elaboration in healthcare simulation and in line with best practices in simulation, and therefore future adjustments may be necessary.

It can be inferred that a good scenario is one that can be reproduced by everyone, so the more complete, practical and useful, the better its application. As elaborating scenarios demands time and financial investments, it is important to evaluate the efficiency and effectiveness of the use of the simulation technique. Thus, it is essential that the scenarios are reproducible so that research with more robust methods can be conducted.

One of the limitations of the *ForPEC* was that it was only available on paper, which limited the registration space (for example). Developing applications that make this form and its manual available, if possible associated with the database which enable recovering and analyzing this data, are possibilities which can contribute to improving and updating the content of this instrument, solving the lack of space for the description of the items and adapting *ForPEC* to the user’s needs (customization) by only allowing items which are relevant to the scenario to be selected.

Considering the extension of the instruments and their textual format, it is pertinent to analyze other methods to reach consensus by the experts which are less labor intensive. Another factor was the greater preponderant participation of nurses in the test. Therefore, including other professional categories could bring different contributions. All these limitations should be considered in future research.

## Conclusion

The Healthcare Simulation Scenario Planning and Elaboration Form (*Formulário de Planejamento e Elaboração de Cenário em Simulação Health Care Simulation–ForPEC)* version 1.3, and its Operational Manual, version 1.2, proved to be valid instruments in terms of their content. The careful selection of experts and their contributions, most of which were incorporated into the instruments, favored their improvement. The feasibility assessment of *ForPEC* and its Manual allowed them to be legitimized as feasible for scenario building by health professionals.

That said, it is recommended to use *ForPEC* and its Operational Manual for planning and elaborating scenarios with the objective of training and qualifying students and health professionals in the necessary skills for their performance, and consequently for patient safety.

## Supporting information

S1 File (PDF)

S2 File (PDF)
