# Supplementary material for: Form for planning and elaborating high fidelity simulation scenarios: A validation study
Source: PLoS One. 2022 Sep 28;17(9):e0274239. doi: 10.1371/journal.pone.0274239 (PMC9518865; doi:10.1371/journal.pone.0274239)
Supplement: S1 File — (PDF) [file pone.0274239.s001.pdf]

PART I: GENERAL PLANNING

1. Scenario:			
2. Institution of the target audience:			
3. Target audience:			
4. Authors responsible for the construction:			
5. Construction date:		6. Revision date:	
7. Pilot test date:		8. Considerations regarding the pilot test:	
9. Instructor(s)/Facilitator(s):			
10. Scenario category, according to the number of specific objectives:	( ) A	( ) B	( ) C

PART II: MINIMUM REQUIREMENTS

11. Pre-simulation knowledge required:	
12. Pre-simulation activities required:	
13. Suggested post-simulation activities:	
14. Pre-simulation evaluation:	
15. Post-simulation evaluation:	

PART III: LEARNING FROM RESULTS

16. General objective(s):	
17. Specific objective(s):	
18. Relevant critical points of the scenario:<br>( ) CRM *(Crew Resource Management)*:<br><br>( ) Others:	
19. Expected results:	
20. Indicators:	
21. Opportunities to improve the scenario:	

Este manual operacional para o preenchimento do formulário de planejamento e elaboração de cenário, versão 1.2 de outubro de 2018 contém informações privilegiadas e/ou confidenciais de propriedade da **SIMSAFETY** Treinamento Desenvolvimento & Educação.
All use (economic or otherwise) depends on prior, formal and express authorization from the author, including partial or complete reproduction and use in any form. When using the document, it is mandatory to mention the name of the person who authored the work."
SIMSAFETY

PART IV: REFERENCES FOR THE PREPARATION OF CONTENT AND SUBMISSION OF THE MATERIAL FOR PRE-OR POST-SIMULATION READING

22. References

PART V: SIMULATION CENTER LOGISTICS

Volunteer information (Student/Participant)					
23. Volunteer(s):					
24. Number of volunteers for the initial scenario:	(1)	(2)	(3)	(4)	(> 4), specify: ______
25. Will the insertion of more volunteers be allowed after beginning the scenario?	( ) No	( ) Yes<br>How many? _______<br><br>Who?_________			

Duration	
26. Duration of instructions	
27. Scenario duration time	
28. Feedback from the standardized actor/actress/patient	
29. Debriefing	

Technological and Human Resources to compose the scenario character	
30. Simulator/Standardized Patient	31. Name of character
( ) High-fidelity simulator	
( ) Medium-fidelity simulator	
( ) Low-fidelity simulator	
( ) Actor/actress	
( ) Hybrid	
( ) Instructor	
( ) Student/Participant	
( ) Patient	

General instructions

Este manual operacional para o preenchimento do formulário de planejamento e elaboração de cenário, versão 1.2 de outubro de 2018 contém informações privilegiadas e/ou confidenciais de propriedade da **SIMSAFETY** Treinamento Desenvolvimento & Educação.
All use (economic or otherwise) depends on prior, formal and express authorization from the author, including partial or complete reproduction and use in any form. When using the document, it is mandatory to mention the name of the person who authored the work."

SIMSAFETY

Simulator/Standardized Patient	32. Select the items to start the scenario	33. Wait outside the scenario	34. Describe the time to enter the scenario location	35. Describe the character's position in the setting for audio and video logistics
( ) High-fidelity simulator				
( ) Medium-fidelity simulator				
( ) Low-fidelity simulator				
( ) Actor/actress				
( ) Hybrid				
( ) Instructor				
( ) Student/Participant				
( ) Patient				
36. Keyword for scenario interruption:				

37. Monitoring	
( ) EKG	( ) ICP
( ) Oximetry	( ) Cardiac Output
( ) NIBP	( ) PAP
( ) IBP	( )
( ) CVP	( )
( ) Temperature	( )
( ) Capnography	( )

38. Accesses	
Catheters / Drains / Dressings	Location and characteristic
( ) Peripheral Venous Access:	
( ) Central Venous Access:	
( ) Intubation:	
( ) Oxygen therapy:	
( ) NG tube:	
( ) NE tube:	
( ) Urinary catheter:	
( ) Chest tube:	

Este manual operacional para o preenchimento do formulário de planejamento e elaboração de cenário, versão 1.2 de outubro de 2018 contém informações privilegiadas e/ou confidenciais de propriedade da **SIMSAFETY** Treinamento Desenvolvimento & Educação.
All use (economic or otherwise) depends on prior, formal and express authorization from the author, including partial or complete reproduction and use in any form. When using the document, it is mandatory to mention the name of the person who authored the work."
SIMSAFETY

( ) JP drain:	
( ) Incisions:	
( ) Dressings:	
( )	
( )	

39. Equipment and Materials		
( ) PPE (glove, mask and glasses)	( ) Identification bracelet	( ) Ventilator
( ) Alcohol gel	( ) Patient's medical record	( ) Aspirator
( ) Stethoscope	( ) Specific forms	( ) Infusion pump
( ) Sphygmomanometer	( ) Intravenous infusions, specify:_______	( ) Emergency vehicle
( ) Thermometer	( ) Medicines, Specify:________	( ) Defibrillator / cardioverter
( ) Tray	( ) Rigid plank	( ) BIPAP
( ) Material venous access	( ) Intubation Material	( ) Scalpel
( ) Gauze	( ) Otoscope	( )
( ) Bandage	( ) Glucometer	( )
( )	( )	( )
( )	( )	( )

40. Ambiance/environment			
Scenario location:			
( ) Stretcher	( ) Telephone		( ) Armchair
( ) Serum support	( ) Computer		( ) Dining table
( ) Accessory table	( ) Cups		( ) Table
( ) Examination Lamp	( ) Water		( ) Chair
( ) Step	( ) Tissue		( ) Others:
Ambient sound:	Configuration:		
( ) Ambulance siren	( ) continuous	( ) Interruption: specify time:___	
( ) Background music	( ) continuous	( ) Interruption: specify time:___	
( ) Beeping from monitors	( ) continuous	( ) Interruption: specify time:___	
( ) Respiratory ventilator	( ) continuous	( ) Interruption: specify time:___	
( ) Crying	( ) continuous	( ) Interruption: specify time: _____	
( ) Others:	( ) continuous	( ) Interruption: specify time:___	

41.Make-up/*Moulage* and Accessories			
Location	Type	Size	Photo

Este manual operacional para o preenchimento do formulário de planejamento e elaboração de cenário, versão 1.2 de outubro de 2018 contém informações privilegiadas e/ou confidenciais de propriedade da **SIMSAFETY** Treinamento Desenvolvimento & Educação.
All use (economic or otherwise) depends on prior, formal and express authorization from the author, including partial or complete reproduction and use in any form. When using the document, it is mandatory to mention the name of the person who authored the work."
SIMSAFETY

PART VI: COMPLETE CASE DESCRIPTION

42.Scenario:	
43. Case description:<br><br>Start of scenario:	

Character History and Anamnesis			
44. Complete name:			
45. Location:			
46. Medical record:		47. Bed:	
48. Weight:	49. Height:	50. Allergies:	
51. Gender: ( ) M ( ) F		52. Date of birth:	
53. Main complaint:			
54. History of current illness:			
55. Family history:			
56. Social history:			
57. Background:			
58. Medication use:			
59. Anamnesis:	General		
	Dermatological		
	Head and neck		
	Nervous		
	Respiratory		
	Cardiovascular		
	Gastrointestinal		
	Urinary		

Este manual operacional para o preenchimento do formulário de planejamento e elaboração de cenário, versão 1.2 de outubro de 2018 contém informações privilegiadas e/ou confidenciais de propriedade da **SIMSAFETY** Treinamento Desenvolvimento & Educação.
All use (economic or otherwise) depends on prior, formal and express authorization from the author, including partial or complete reproduction and use in any form. When using the document, it is mandatory to mention the name of the person who authored the work."
SIMSAFETY

	Genital	
	Skeletal muscle	
	Limbs/Peripheral Vascular	
	Psychological	
60. Previous exams performed		

Technical information		
61. Monitor: ( ) Off and not connected or<br>( ) Powered on and connected to:<br>62. ( ) HR: ______<br>63. ( ) Initial cardiac rhythm: ______<br>64. ( ) BP: ______<br>65. ( ) RR: ______<br>66. ( ) Temp.: ______<br>67. ( ) Sat O_2: ______<br>68. ( ) CVP: ______<br>69: ( ) MAP: ______<br>70. ( ) Other parameters:__________		
71. CFT:	72. Capillary Glycemia:	73.Glasgow<br>(E V M)

Character Profile Description		
74.Psychological:		
75. Social:		
76. Physical:		
77. Costume:		
78. Technical:		
Scenario evolution		
79. Attitude A (Positive outcome)	81. Attitude B (Negative outcome)	
80. Phrases which can be used	82. Phrases which can be used	

Este manual operacional para o preenchimento do formulário de planejamento e elaboração de cenário, versão 1.2 de outubro de 2018 contém informações privilegiadas e/ou confidenciais de propriedade da **SIMSAFETY** Treinamento Desenvolvimento & Educação.
All use (economic or otherwise) depends on prior, formal and express authorization from the author, including partial or complete reproduction and use in any form. When using the document, it is mandatory to mention the name of the person who authored the work."
SIMSAFETY

83. Scenario evolution (Vital and Clinical Parameters)						
Condition	1	2	3	4	5	6*
Clinical						
Level of consciousness						
Heart rate						
Cardiac rhythm						
BP						
RR						
Sat O2						
Temperature						
etCO2						
Others:						
Actions and transitions						

*Add columns if necessary

PART VII: EXAM RESULTS

84. Laboratory or Reports			
Exams	Initial result	Final result	Normality values
Sodium			
Potassium			
Calcium			
Magnesium			
Hb			
Hematocrit			
Platelets			
Ultrasound			
Others:			

85. Images	

Este manual operacional para o preenchimento do formulário de planejamento e elaboração de cenário, versão 1.2 de outubro de 2018 contém informações privilegiadas e/ou confidenciais de propriedade da **SIMSAFETY** Treinamento Desenvolvimento & Educação.
All use (economic or otherwise) depends on prior, formal and express authorization from the author, including partial or complete reproduction and use in any form. When using the document, it is mandatory to mention the name of the person who authored the work."
SIMSAFETY

PART VIII: DEBRIEFING

86. Audio Video			
( ) Individual	( ) Group	( ) With video	( ) No video
87.Objective(s)			
88.Suggested questions for the Debriefing			
89. Critical points			

PART IX: CHECK LIST/SKILLS PERFORMANCE ANALYSIS

90.Check List/Skills performance analysis		
**Scenario**		
**Topics**	**Actions**	**"X" for the performed actions**
**Technical skills**		

Este manual operacional para o preenchimento do formulário de planejamento e elaboração de cenário, versão 1.2 de outubro de 2018 contém informações privilegiadas e/ou confidenciais de propriedade da **SIMSAFETY** Treinamento Desenvolvimento & Educação.
All use (economic or otherwise) depends on prior, formal and express authorization from the author, including partial or complete reproduction and use in any form. When using the document, it is mandatory to mention the name of the person who authored the work."
SIMSAFETY

**Patient and Employee Safety**		
**Non-technical skills**		

Additional comments:

PART X: CASE READING TO PARTICIPANT

91. case reading to participant – Scenario:
**Unit:**<br><br>**Professional:**<br><br>**Case summary:**<br><br><u>Scenario start:</u>

Este manual operacional para o preenchimento do formulário de planejamento e elaboração de cenário, versão 1.2 de outubro de 2018 contém informações privilegiadas e/ou confidenciais de propriedade da **SIMSAFETY** Treinamento Desenvolvimento & Educação.
All use (economic or otherwise) depends on prior, formal and express authorization from the author, including partial or complete reproduction and use in any form. When using the document, it is mandatory to mention the name of the person who authored the work."
SIMSAFETY

Scenario Agenda

Este manual operacional para o preenchimento do formulário de planejamento e elaboração de cenário, versão 1.2 de outubro de 2018 contém informações privilegiadas e/ou confidenciais de propriedade da **SIMSAFETY** Treinamento Desenvolvimento & Educação.
All use (economic or otherwise) depends on prior, formal and express authorization from the author, including partial or complete reproduction and use in any form. When using the document, it is mandatory to mention the name of the person who authored the work."

SIMSAFETY
