# Supplementary material for: Form for planning and elaborating high fidelity simulation scenarios: A validation study
Source: PLoS One. 2022 Sep 28;17(9):e0274239. doi: 10.1371/journal.pone.0274239 (PMC9518865; doi:10.1371/journal.pone.0274239)
Supplement: S2 File — (PDF) [file pone.0274239.s002.pdf]

OPERATIONAL MANUAL FOR COMPLETING THE PLANNING FORM AND PREPARING A REALISTIC HEALTH SIMULATION SCENARIO (ForPEC)

Version 1.2 (October 2018)

SÃO PAULO

2018

Este manual operacional para o preenchimento do formulário de planejamento e elaboração de cenário, versão 1.2 de outubro de 2018 contém informações privilegiadas e/ou confidenciais de propriedade da **SIMSAFETY** Treinamento Desenvolvimento & Educação.
All use (economic or otherwise) depends on prior, formal and express authorization from the author, including partial or complete reproduction and use in any form. When using the document, it is mandatory to mention the name of the person who authored the work."

SIMSAFETY

OPERATIONAL MANUAL FOR COMPLETING THE PLANNING FORM AND PREPARING A REALISTIC HEALTH SIMULATION SCENARIO (ForPEC)

Versão 1.2 (outubro 2018)

PREPARED BY: REGINA MAYUMI UTIYAMA KANEKO

Este manual operacional para o preenchimento do formulário de planejamento e elaboração de cenário, versão 1.2 de outubro de 2018 contém informações privilegiadas e/ou confidenciais de propriedade da **SIMSAFETY** Treinamento Desenvolvimento & Educação.
All use (economic or otherwise) depends on prior, formal and express authorization from the author, including partial or complete reproduction and use in any form. When using the document, it is mandatory to mention the name of the person who authored the work."

SIMSAFETY

INDEX

INTRODUCTION 4
PART I: GENERAL PLANNING 5
PART II: MINIMUM REQUIREMENTS 5
PART III: LEARNING FROM RESULTS 6
PART IV: REFERENCES FOR THE PREPARATION OF CONTENT AND SUBMISSION OF THE MATERIAL FOR PRE-OR POST-SIMULATION READING 7
PART V: SIMULATION CENTER LOGISTICS 7
PART VI: COMPLETE CASE DESCRIPTION 11
PART VII: EXAM RESULTS 15
PART VIII: DEBRIEFING 16
PART IX: CHECK LIST/SKILLS PERFORMANCE ANALYSIS 17
PART X: CASE READING TO PARTICIPANT 18
ANNEX 20
  Annex 1 Technical scenario example 20
  Annex 2 Example of a behavioral scenario 32
REFERENCES 44

Este manual operacional para o preenchimento do formulário de planejamento e elaboração de cenário, versão 1.2 de outubro de 2018 contém informações privilegiadas e/ou confidenciais de propriedade da **SIMSAFETY** Treinamento Desenvolvimento & Educação.
All use (economic or otherwise) depends on prior, formal and express authorization from the author, including partial or complete reproduction and use in any form. When using the document, it is mandatory to mention the name of the person who authored the work."
SIMSAFETY

INTRODUCTION

This operational manual for completing the Realistic Health Simulation Planning and Development Form (ForPEC), version 1.2 of October 2018, aims to serve as a reference source for the construction of health simulation scenarios using ForPEC, version October 1.3, 2018.

It is organized into parts according to the numbering in ForPEC, version 1.3 October 2018 and with a detailed description of each item below.

A technical scenario and a behavioral scenario are included in the annex as examples to facilitate understanding and use of this manual.

Este manual operacional para o preenchimento do formulário de planejamento e elaboração de cenário, versão 1.2 de outubro de 2018 contém informações privilegiadas e/ou confidenciais de propriedade da **SIMSAFETY** Treinamento Desenvolvimento & Educação.
All use (economic or otherwise) depends on prior, formal and express authorization from the author, including partial or complete reproduction and use in any form. When using the document, it is mandatory to mention the name of the person who authored the work."
SIMSAFETY

PART I: GENERAL PLANNING

1. Scenario:			
2. Institution of the target audience:			
3. Target audience:			
4. Authors responsible for the construction:			
5. Construction date:		6. Revision date:	
7. Pilot test date:		8. Considerations regarding the pilot test:	
9. Instructor(s)/Facilitator(s):			
10. Scenario category, according to the number of specific objectives:	( ) A	( ) B	( ) C

1. Register the scenario title.
2. Register the target audience institution.
3. Determine the target audience of the scenario, understand who the participants are to plan and focus the scenario actions.
4. Register the authors responsible for building the scenario.
5. Record the scenario construction date.
6. Record the scenario review date. The version may change according to the modifications and additions made to update and change the content.
7. Insert the date when the scenario pilot was performed, meaning when the scenario was tested.
8. Describe the result of the scenario pilot: Positive points and critical points that promoted its modification and additions to the scenario.
9. Record the name(s) of the scenario instructor(s)/facilitator(s).
10. Categorize the scenario according to the number of objectives:
"A" for a specific learning objective;
"B" for two specific learning objectives; and
"C" for more than two specific learning objectives.

PART II: MINIMUM REQUIREMENTS

11. Pre-simulation knowledge required:	
12. Pre-simulation activities required:	
13. Suggested post-simulation activities:	
14. Pre-simulation evaluation:	
15. Post-simulation evaluation:	

11. Record the necessary prior knowledge prior to performing the scenario, such as pharmacology, physiology, nursing diagnosis, radiology and imaging, etc.

Este manual operacional para o preenchimento do formulário de planejamento e elaboração de cenário, versão 1.2 de outubro de 2018 contém informações privilegiadas e/ou confidenciais de propriedade da **SIMSAFETY** Treinamento Desenvolvimento & Educação.
All use (economic or otherwise) depends on prior, formal and express authorization from the author, including partial or complete reproduction and use in any form. When using the document, it is mandatory to mention the name of the person who authored the work."
SIMSAFETY

12. Record the necessary teaching and learning activities prior to the realization of the scenario, such as reading articles, lectures, e-learning, monitored practice and others.
13. Record the content transfer activities to be developed after the scenario is carried out, such as articles, guidelines, reminders, critical points of the scenario where students/participants had greater difficulty in conducting them and others. These content transfer activities are planned in line with the target audience, the results and indicators selected for the continuity of learning.
14. Determine the performance of the student/participant assessment prior to the realization of the scenario. Describe the strategy to be used for this assessment, such as: multiple choice test, essay questions, procedure validation, OSCE (Objective Structured Clinical Examination) and others.
15. Determine the performance of student/participant assessment after the scenario. Describe the strategy to be used for this assessment, such as: multiple choice test, essay questions, procedure validation, OSCE (Objective Structured Clinical Examination) and others.

Note: Content transfer activities, pre and post simulation assessments are not mandatory. Each institution should outline its guidelines.

PART III: LEARNING FROM RESULTS

16. General objective(s):	
17. Specific objective(s):	
18. Relevant critical points of the scenario:<br>( ) CRM *(Crew Resource Management)*:<br><br>( ) Others:	
19. Expected results:	
20. Indicators:	
21. Opportunities to improve the scenario:	

16. List the general objective(s) of the scenario.
17. List the specific objective(s) of the scenario.

Este manual operacional para o preenchimento do formulário de planejamento e elaboração de cenário, versão 1.2 de outubro de 2018 contém informações privilegiadas e/ou confidenciais de propriedade da **SIMSAFETY** Treinamento Desenvolvimento & Educação.
All use (economic or otherwise) depends on prior, formal and express authorization from the author, including partial or complete reproduction and use in any form. When using the document, it is mandatory to mention the name of the person who authored the work."
SIMSAFETY

18. Describe the relevant critical points of the scenario to guide the scenario elaboration and debriefing. When selecting the CRM (Crew Resource Management), list the main key points of the scenario: leadership, communication, teamwork, situational awareness, decision making and use of all available resources. For the other critical points, specify, for example, the update of some protocol item, “TeamSTEEPS”, “ANTS”, “Ottawa GRS”, etc.
19. Establish the expected results in the scenario, for example, satisfaction of the participants, achievement of the objective proposed in the scenario, etc.
20. Describe the organizational indicator(s) for monitoring, eg urinary infection rate after scenarios related to the topic.
21. After completing the scenario, fill in the positive points and opportunities to improve the scenario, for example, in the training/course, the need to specify, in addition to the fasting time, the type of food eaten at the last meal or to add materials, was identified, equipment and exams.

PART IV: REFERENCES FOR THE PREPARATION OF CONTENT AND SUBMISSION OF THE MATERIAL FOR PRE-OR POST-SIMULATION READING

22. References

22. List all references used to prepare the scenario (articles, guidelines, policies and institutional routines).

PART V: SIMULATION CENTER LOGISTICS

Volunteer information (Student/Participant)					
23. Volunteer(s):					
24. Number of volunteers for the initial scenario:	(1)	(2)	(3)	(4)	(> 4), specify: ______
25. Will the insertion of more volunteers be allowed after beginning the scenario?	( ) No	( ) Yes<br>How many? _______<br><br>Who?__________			

23. Determine the volunteer(s) in the scenario, specify the professional category and function.
24. Tick the number of volunteers to start the scenario. Specify the number if it exceeds four volunteers.
25. Mark the insertion of other volunteer(s) after beginning the scenario. If yes, describe the number and who are the professionals.

Duration

Este manual operacional para o preenchimento do formulário de planejamento e elaboração de cenário, versão 1.2 de outubro de 2018 contém informações privilegiadas e/ou confidenciais de propriedade da **SIMSAFETY** Treinamento Desenvolvimento & Educação.
All use (economic or otherwise) depends on prior, formal and express authorization from the author, including partial or complete reproduction and use in any form. When using the document, it is mandatory to mention the name of the person who authored the work."
SIMSAFETY

26. Duration of instructions	
27. Scenario duration time	
28. Feedback from the standardized actor/actress/patient	
29. Debriefing	

26. Determine the duration time in minutes for carrying out the orientations to the volunteer(s). These guidelines include case reading, scenario location, scenario context and setting, and simulator/robot functionality.
27. Determine the duration time in minutes for the realization of the scenario.
28. Determine the duration in minutes for the actor/actress' feedback if the practice is established as a routine in the institution. Otherwise, enter “NA” (not applicable)
29. Determine the duration time in minutes for the debriefing.

Technological and Human Resources to compose the scenario character	
30. Simulator/Standardized Patient	31. Name of character
( ) High-fidelity simulator	
( ) Medium-fidelity simulator	
( ) Low-fidelity simulator	
( ) Actor/actress	
( ) Hybrid	
( ) Instructor	
( ) Student/Participant	
( ) Patient	

30. Select the technological resource(s): simulators and robots and the human resource(s) to compose the character of the scenario.
- ✓ Low-fidelity simulator: static simulators for reproducing procedures and techniques.
- ✓ Medium-fidelity simulator: simulators that replicate some functions, such as cardiac, pulmonary, abdominal auscultation and the presence of a pulse.
- ✓ High-fidelity simulator: high complexity simulators which enable replicating the physiological functions of the human being.
  The standardized patient refers to the people who simulate the character of the scenario.

31. Describe the full name of the character(s).

General instructions				
Simulator/Standardized Patient	32. Select the items	33. Wait outside the scenario	34. Describe the time to enter the	35. Describe the character's

Este manual operacional para o preenchimento do formulário de planejamento e elaboração de cenário, versão 1.2 de outubro de 2018 contém informações privilegiadas e/ou confidenciais de propriedade da **SIMSAFETY** Treinamento Desenvolvimento & Educação.
All use (economic or otherwise) depends on prior, formal and express authorization from the author, including partial or complete reproduction and use in any form. When using the document, it is mandatory to mention the name of the person who authored the work."
SIMSAFETY

	to start the scenario		scenario location	position in the setting for audio and video logistics
( ) High-fidelity simulator				
( ) Medium-fidelity simulator				
( ) Low-fidelity simulator				
( ) Actor/actress				
( ) Hybrid				
( ) Instructor				
( ) Student/Participant				
( ) Patient				
36. Keyword for scenario interruption:				

32. Mark with an (x) the characters that make up the scenario at the beginning of the activity.
33. Mark with an (x) if the character does not start the scenario at the location and fill in item 34.
34. Determine the time in minutes and/or seconds for the character to enter the scenario location.
35. Determine the initial positioning and its evolution in the scenario for the best audio and video capture.
36. Select and record a keyword to consider immediately stopping the scenario for student/participant and character safety issues.

37. Monitoring	
( ) EKG	( ) ICP
( ) Oximetry	( ) Cardiac Output
( ) NIBP	( ) PAP
( ) IBP	( )
( ) CVP	( )
( ) Temperature	( )
( ) Capnography	( )

37. Select all monitoring features and add others for scenario driving if necessary.

38. Accesses	
Catheters / Drains / Dressings	Location and characteristic

Este manual operacional para o preenchimento do formulário de planejamento e elaboração de cenário, versão 1.2 de outubro de 2018 contém informações privilegiadas e/ou confidenciais de propriedade da **SIMSAFETY** Treinamento Desenvolvimento & Educação.
All use (economic or otherwise) depends on prior, formal and express authorization from the author, including partial or complete reproduction and use in any form. When using the document, it is mandatory to mention the name of the person who authored the work."
SIMSAFETY

( ) Peripheral Venous Access:	
( ) Central Venous Access:	
( ) Intubation:	
( ) Oxygen therapy:	
( ) NG tube:	
( ) NE tube:	
( ) Urinary catheter:	
( ) Chest tube:	
( ) JP drain:	
( ) Incisions:	
( ) Dressings:	
( )	
( )	

38. Select all accesses (catheter, drain, dressing, etc.), their location and characteristic for beginning the scenario. Add other items if needed.

39. Equipment and Materials		
( ) PPE (glove, mask and glasses)	( ) Identification bracelet	( ) Ventilator
( ) Alcohol gel	( ) Patient's medical record	( ) Aspirator
( ) Stethoscope	( ) Specific forms	( ) Infusion pump
( ) Sphygmomanometer	( ) Intravenous infusions, specify:______	( ) Emergency vehicle
( ) Thermometer	( ) Medicines, Specify:_______	( ) Defibrillator / cardioverter
( ) Tray	( ) Rigid plank	( ) BIPAP
( ) Material venous access	( ) Intubation Material	( ) Scalpel
( ) Gauze	( ) Otoscope	( )
( ) Bandage	( ) Glucometer	( )
( )	( )	( )
( )	( )	( )

39. Tick all the equipment and materials needed to compose the scenario and add, if necessary.

40. Ambiance/environment		
Scenario location:		
( ) Stretcher	( ) Telephone	( ) Armchair
( ) Serum support	( ) Computer	( ) Dining table
( ) Accessory table	( ) Cups	( ) Table
( ) Examination Lamp	( ) Water	( ) Chair

Este manual operacional para o preenchimento do formulário de planejamento e elaboração de cenário, versão 1.2 de outubro de 2018 contém informações privilegiadas e/ou confidenciais de propriedade da **SIMSAFETY** Treinamento Desenvolvimento & Educação.
All use (economic or otherwise) depends on prior, formal and express authorization from the author, including partial or complete reproduction and use in any form. When using the document, it is mandatory to mention the name of the person who authored the work."
SIMSAFETY

( ) Step	( ) Tissue	( ) Others:
Ambient sound:	Configuration:	
( ) Ambulance siren	( ) continuous	( ) Interruption: specify time:___
( ) Background music	( ) continuous	( ) Interruption: specify time:___
( ) Beeping from monitors	( ) continuous	( ) Interruption: specify time:___
( ) Respiratory ventilator	( ) continuous	( ) Interruption: specify time:___
( ) Crying	( ) continuous	( ) Interruption: specify time: _____
( ) Others:	( ) continuous	( ) Interruption: specify time:___

40. Describe the location of the scenario, for example, room, ward, intensive care unit, home, office, reception, others. Select all the furniture and accessories necessary for the setting of the scenario and add, if necessary. Select ambient sound and its setting.

41.Make-up/*Moulage* and Accessories			
Location	Type	Size	Photo

41. Describe the location, type and size of makeup/moulage, both on the simulator and on the actor/actress. Describe the use of accessories such as amputated leg, mastectomy, pregnancy and other devices. Include photo if available.

PART VI: COMPLETE CASE DESCRIPTION

42.Scenario:	
43. Case description:<br><br>Start of scenario:	

42. Register the scenario title.
43. Describe the complete scenario case and the beginning of the scenario to guide the facilitators/trainers in conducting the scenario. The reading of the scenario by the students/participants is described in item 91 (Reading the case) of the form. In this case, the description of the complete case may be similar or amended to only provide the information necessary to start conducting the scenario.

Este manual operacional para o preenchimento do formulário de planejamento e elaboração de cenário, versão 1.2 de outubro de 2018 contém informações privilegiadas e/ou confidenciais de propriedade da **SIMSAFETY** Treinamento Desenvolvimento & Educação.
All use (economic or otherwise) depends on prior, formal and express authorization from the author, including partial or complete reproduction and use in any form. When using the document, it is mandatory to mention the name of the person who authored the work."
SIMSAFETY

Character History and Anamnesis		
44. Complete name:		
45. Location:		
46. Medical record:		47. Bed:
48. Weight:	49. Height:	50. Allergies:
51. Gender: ( ) M ( ) F		52. Date of birth:
53. Main complaint:		
54. History of current illness:		
55. Family history:		
56. Social history:		
57. Background:		
58. Medication use:		
59. Anamnesis:	General	
	Dermatological	
	Head and neck	
	Nervous	
	Respiratory	
	Cardiovascular	
	Gastrointestinal	
	Urinary	
	Genital	
	Skeletal muscle	
	Limbs/Peripheral Vascular	
	Psychological	
60. Previous exams performed		

44. Register the character’s full name.
45. Record the location of the scenario.
46. Allocate a character’s medical record.
47. Allocate a character bed number.
48. Record the character’s weight in kg.
49. Record the height in cm of the character.
50. Record the character’s allergies.

Este manual operacional para o preenchimento do formulário de planejamento e elaboração de cenário, versão 1.2 de outubro de 2018 contém informações privilegiadas e/ou confidenciais de propriedade da **SIMSAFETY** Treinamento Desenvolvimento & Educação.
All use (economic or otherwise) depends on prior, formal and express authorization from the author, including partial or complete reproduction and use in any form. When using the document, it is mandatory to mention the name of the person who authored the work."
SIMSAFETY

51. Tick M for male and F for female.
52. Record the character’s date of birth.
53. Record the character’s main complaint.
54. Record current illness history.
55. Record the character’s family history.
56. Record the character’s social history. Social history refers to financial conditions, personal interests, professional activities and social relationships that can interfere with the conduct of the scenario, for example, low income makes it difficult to adhere to a diet guided by the student/participant.
57. Record the character’s background.
58. Register medications in use.
59. Describe the findings on the systems in the anamnesis.
60. List the previous exams performed and their value to be considered in the scenario.
Note: Insert “NA” when not applicable in the case of the scenario.

<table>
<tr><th colspan="3">Technical information</th></tr>
<tr><td colspan="3">61. Monitor: ( ) Off and not connected or
( ) Powered on and connected to:
62. ( ) HR: ______
63. ( ) Initial cardiac rhythm: ______
64. ( ) BP: ______
65. ( ) RR: ______
66. ( ) Temp.: ______
67. ( ) Sat O_2: ______
68. ( ) CVP: ______
69: ( ) MAP: ______
70. ( ) Other parameters:__________</td></tr>
<tr><td>71. CFT:</td><td>72. Capillary Glycemia:</td><td>73.Glasgow
(E V M)</td></tr>
</table>

61. Select the monitor’s initial condition according to the scenario description. When choosing on and connected, select the items that will appear on the monitor and their respective parameters. Continuing with the parameter changes, complete item 83 (scenario evolution).
62. Record starting HR (heart rate).
63. Determine the initial cardiac rhythm.
64. Record initial BP (blood pressure).
65. Record the initial RR (respiratory rate).
66. Record the Start Temperature.

Este manual operacional para o preenchimento do formulário de planejamento e elaboração de cenário, versão 1.2 de outubro de 2018 contém informações privilegiadas e/ou confidenciais de propriedade da **SIMSAFETY** Treinamento Desenvolvimento & Educação.
All use (economic or otherwise) depends on prior, formal and express authorization from the author, including partial or complete reproduction and use in any form. When using the document, it is mandatory to mention the name of the person who authored the work."
SIMSAFETY

67. Record the initial O_2 Saturation
68. Record the initial CVP (central venous pressure).
69. Record baseline MAP (mean arterial pressure).
70. List other necessary parameters according to the case description.
71. Record the CFT (Capillary Filling Time).
72. Record the capillary blood glucose value.
73. Register the initial Glasgow assessment.

Character Profile Description		
74.Psychological:		
75. Social:		
76. Physical:		
77. Costume:		
78. Technical:		
Scenario evolution		
79. Attitude A (Positive outcome)		81. Attitude B (Negative outcome)
80. Phrases which can be used		82. Phrases which can be used

74. Describe the character’s psychological profile.
75. Describe the character’s social profile, for example, unemployed for 1 year, dependent on the mother's retirement, unable to follow the recommended diet and take the medicine prescribed by the doctor.
76. Describe the character’s physical profile, for example, obese, age 45, emaciated, etc.
77. Describe the costumes needed to compose the character’s case.
78. Describe the technical components to assist the character in approaching a technical scenario, for example, signs and symptoms of a stroke patient, patient care flow in the laboratory, “SPIKES” protocol in communicating bad news, etc.
79. Determine the evolution of the scenario to the positive outcome, according to the performance of the student/participant.
80. Develop phrases for the character to use at the beginning, in the evolution and at the end of the scenario for the positive outcome.
81. Determine the evolution of the scenario to a negative outcome, according to student/participant performance.
82. Develop phrases for the character to use at the beginning, in the evolution and at the end of the scenario for the negative outcome.

Este manual operacional para o preenchimento do formulário de planejamento e elaboração de cenário, versão 1.2 de outubro de 2018 contém informações privilegiadas e/ou confidenciais de propriedade da **SIMSAFETY** Treinamento Desenvolvimento & Educação.
All use (economic or otherwise) depends on prior, formal and express authorization from the author, including partial or complete reproduction and use in any form. When using the document, it is mandatory to mention the name of the person who authored the work."
SIMSAFETY

83. Scenario evolution (Vital and Clinical Parameters)						
Condition	1	2	3	4	5	6*
Clinical						
Level of consciousness						
Heart rate						
Cardiac rhythm						
BP						
RR						
Sat O2						
Temperature						
etCO2						
Others:						
Actions and transitions						

*Add columns if necessary

83. Record the vital and clinical parameters and the expected actions for the transitions and evolution of the patient’s next clinical condition, including a time interval when applicable, according to the conduct and decision-making of the student/participant. Record the duration of each evolution, if applicable, and/or the action that will trigger the next phase of the scenario. These parameters must be recorded and described whenever necessary to conduct the scenario. It is important to develop an algorithm of options for both correct and incorrect behaviors. Other parameters can be entered as needed. Add the number of conditions necessary for the evolution of the scenario.

PART VII: EXAM RESULTS

84. Laboratory or Reports			
Exams	Initial result	Final result	Normality values
Sodium			
Potassium			
Calcium			
Magnesium			

Este manual operacional para o preenchimento do formulário de planejamento e elaboração de cenário, versão 1.2 de outubro de 2018 contém informações privilegiadas e/ou confidenciais de propriedade da **SIMSAFETY** Treinamento Desenvolvimento & Educação.
All use (economic or otherwise) depends on prior, formal and express authorization from the author, including partial or complete reproduction and use in any form. When using the document, it is mandatory to mention the name of the person who authored the work."
SIMSAFETY

Hb			
Hematocrit			
Platelets			
Ultrasound			
Others:			

84. Record the values of the results of laboratory tests or initial and sequential reports to the clinical condition, if applicable. Note the normality value of each recorded exam. Add other tests as needed.

85. Images	

85. Insert the necessary images to conduct the scenario.

PART VIII: DEBRIEFING

86. Audio Video			
( ) Individual	( ) Group	( ) With video	( ) No video
87.Objective(s)			
88.Suggested questions for the Debriefing			
89. Critical points			

86. Select the resource used for the scenario recording. This information is important for the instructor at the time of the debriefing for the purpose of retrieving the image and noting the exact times for the presentation of a specific action. Behavioral aspects and specific techniques can be captured in individual recordings and team actions in group images when recordings are used.
Tick the required individual or group audio and video resources. This information is important for planning, allocating technological resources in scenarios and positioning cameras for the logistics of a simulation center/laboratory.
87. Convey the stated objectives to the scenario.
88. Elaborate questions relevant to the scenario.
89. Take the critical points of the scenario to the debriefing moment in a positive way.

Este manual operacional para o preenchimento do formulário de planejamento e elaboração de cenário, versão 1.2 de outubro de 2018 contém informações privilegiadas e/ou confidenciais de propriedade da **SIMSAFETY** Treinamento Desenvolvimento & Educação.
All use (economic or otherwise) depends on prior, formal and express authorization from the author, including partial or complete reproduction and use in any form. When using the document, it is mandatory to mention the name of the person who authored the work."
SIMSAFETY

PART IX: CHECK LIST/SKILLS PERFORMANCE ANALYSIS

90.Check List/Skills performance analysis		
**Scenario**		
**Topics**	**Actions**	**"X" for the performed actions**
**Technical skills**		
**Patient and Employee Safety**		
**Non-technical skills**		

Additional comments:

90. Develop the checklist/performance analysis of scenario monitoring skills for students/participants divided into technical skills, patient and employee safety, non-technical skills. Exclude if you do not use all components of the checklist.

Este manual operacional para o preenchimento do formulário de planejamento e elaboração de cenário, versão 1.2 de outubro de 2018 contém informações privilegiadas e/ou confidenciais de propriedade da **SIMSAFETY** Treinamento Desenvolvimento & Educação.
All use (economic or otherwise) depends on prior, formal and express authorization from the author, including partial or complete reproduction and use in any form. When using the document, it is mandatory to mention the name of the person who authored the work."
SIMSAFETY

Its use is optional, according to the routine activities of each simulation center or skills laboratory.

PART X: CASE READING TO PARTICIPANT

91. case reading to participant – Scenario:
**Unit:**<br><br>**Professional:**<br><br>**Case summary:**<br><br>Scenario start:<br><br>**Scenario Agenda**

91. Prepare the case reading for the student/volunteer participant of the scenario with the information determined in items 26 (duration time of guidelines), 27 (duration time of the scenario), 28 (Feedback from the actor/actress/standardized patient), and 29 (Debriefing) for the scenario agenda and items 42 (scenario) and 43 (scenario description) for the scenario description. It is an optional item and must be used according to the routine of each service.

In this item (case reading), describe the information that will be made available to students/participants according to the proposed objectives. This description may be different from the description of the case prepared in item 43 (Description of the case).

Este manual operacional para o preenchimento do formulário de planejamento e elaboração de cenário, versão 1.2 de outubro de 2018 contém informações privilegiadas e/ou confidenciais de propriedade da **SIMSAFETY** Treinamento Desenvolvimento & Educação.
All use (economic or otherwise) depends on prior, formal and express authorization from the author, including partial or complete reproduction and use in any form. When using the document, it is mandatory to mention the name of the person who authored the work."
SIMSAFETY

Este manual operacional para o preenchimento do formulário de planejamento e elaboração de cenário, versão 1.2 de outubro de 2018 contém informações privilegiadas e/ou confidenciais de propriedade da **SIMSAFETY** Treinamento Desenvolvimento & Educação.
All use (economic or otherwise) depends on prior, formal and express authorization from the author, including partial or complete reproduction and use in any form. When using the document, it is mandatory to mention the name of the person who authored the work."

ANNEX

Annex 1 Technical scenario example

PART I: GENERAL PLANNING

32. Scenario:	Chest drain		
33. Institution of the target audience:		XXX	
34. Target audience:		Nursing team	
35. Authors responsible for the construction:		Regina Mayumi Utiyama Kaneko	
36. Construction date:	07/05/2016	37. Revision date:	15/05/2016
38. Pilot test date:	14/04/2016	39. Considerations regarding the pilot test:	
40. Instructor(s)/Facilitator(s):		Regina Mayumi Utiyama Kaneko	

41. Scenario category, according to the number of specific objectives:	( ) A	(x) B	( ) C

PART II: MINIMUM REQUIREMENTS

42. Pre-simulation knowledge required:	Anatomy, Physiology of the respiratory system
43. Pre-simulation activities required:	Dialogued expository class
44. Suggested post-simulation activities:	Case Study Discussion (Four Corners Technique)
45. Pre-simulation evaluation:	Reminder Project
46. Post-simulation evaluation:	Written assessment with multiple-choice tests

PART III: LEARNING FROM RESULTS

47. General objective(s):	Discuss nursing care for patients with chest tubes
48. Specific objective(s):	Identify the “clamping” of the chest drain;
49. Relevant critical points of the scenario:<br>50. (x) CRM *(Crew Resource Management)*:<br><br>(x) Others:	 Call for help<br>Communication<br>Use all information<br>Repeatedly re-evaluate<br>set priorities<br><br>Identify drain “clamping”

Este manual operacional para o preenchimento do formulário de planejamento e elaboração de cenário, versão 1.2 de outubro de 2018 contém informações privilegiadas e/ou confidenciais de propriedade da **SIMSAFETY** Treinamento Desenvolvimento & Educação.

All use (economic or otherwise) depends on prior, formal and express authorization from the author, including partial or complete reproduction and use in any form. When using the document, it is mandatory to mention the name of the person who authored the work."

SIMSAFETY

	Evaluation<br>Ethics and professionalism
51. Expected results:	Average of the highest evaluation grade in the evaluation after the simulation;<br>Increase in the rate of knowledge acquisition.
52. Indicators:	Knowledge acquisition rate<br>Average of the pre-simulation assessment grade<br>Post-simulation evaluation grade average<br>Notifications of incidents related to drain “clamping”.
53. Opportunities to improve the scenario:	Professional ethics issues were included as discussions arose in relation to the patient regarding the mistakes committed

PART IV: REFERENCES FOR THE PREPARATION OF CONTENT AND SUBMISSION OF THE MATERIAL FOR PRE-OR POST-SIMULATION READING

54. References
Cipriano FG, Dessote LU. Drenagem pleural. Medicina Ribeirão Preto. 2011;44(1):70-8

PART V: SIMULATION CENTER LOGISTICS

Volunteer information (Student/Participant)					
55. Volunteer(s):	Nursing technician				
56. Number of volunteers for the initial scenario:	(1)	(2)	(3)	(4)	(>4), specify: ______
57. Will the insertion of more volunteers be allowed after beginning the scenario?	( ) No	(x) Yes How many? 01<br>Who? Nurse			

Duration	
58. Duration of instructions	5 minutes
59. Scenario duration time	10 minutes
60. Feedback from the standardized actor/actress/patient	5 minutes

Este manual operacional para o preenchimento do formulário de planejamento e elaboração de cenário, versão 1.2 de outubro de 2018 contém informações privilegiadas e/ou confidenciais de propriedade da **SIMSAFETY** Treinamento Desenvolvimento & Educação.
All use (economic or otherwise) depends on prior, formal and express authorization from the author, including partial or complete reproduction and use in any form. When using the document, it is mandatory to mention the name of the person who authored the work."
SIMSAFETY

61. Debriefing	20 minutes

Technological and Human Resources to compose the scenario character	
62. Simulator/Standardized patient	63. Name of character
( ) High-fidelity simulator	
(x) Medium-fidelity simulator	Pedro da Silva
( ) Low-fidelity simulator	
( ) Actor/actress	
( ) Hybrid	
( ) Instructor	
( ) Student/Participant	
( ) Patient	

General instructions				
Simulator/Standardized patient	32. Select the items to start the scenario	33. Wait outside the scenario	34. Describe the time to enter the scenario location	35.Describe the character's position in the setting for audio and video logistics
( ) High-fidelity simulator				
(x) Medium-fidelity simulator	x			Position the simulator so that when the volunteer identifies the drain "clamping", the camera records this moment
( ) Low-fidelity simulator				
( ) Actor/actress				
( ) Hybrid				
( ) Instructor				
( ) Student/Participant				
( ) Patient				
36.Keyword for scenario interruption:	Europe			

Este manual operacional para o preenchimento do formulário de planejamento e elaboração de cenário, versão 1.2 de outubro de 2018 contém informações privilegiadas e/ou confidenciais de propriedade da **SIMSAFETY** Treinamento Desenvolvimento & Educação.
All use (economic or otherwise) depends on prior, formal and express authorization from the author, including partial or complete reproduction and use in any form. When using the document, it is mandatory to mention the name of the person who authored the work."
SIMSAFETY

37. Monitoring	
(x) EKG	( ) ICP
(x) Oximetry	( ) Cardiac Output
(x) NIBP	( ) PAP
( ) IBP	( )
( ) CVP	( )
(x) Temperature	( )
( ) Capnography	( )

38. Accesses	
Catheters/Drains/Dressings	Location and characteristic
(x) Peripheral venous access:	Peripheral venous access permeabilized in the right upper limb
( ) Central venous access:	
( ) Intubation:	
( ) Oxygen therapy:	
( ) NG tube:	
( ) NE tube:	
( ) Urinary catheter:	
(x) Chest drain:	Drain vial with bloody secretion in HTD, flow rate of 50 mL. Dressing.
( ) JP drain:	
(x) Incisions:	Right anterolateral transverse thoracic region
(x) Dressings:	Occlusive dressing in the right anterolateral transverse thoracic region
( )	
( )	

39.Equipment and Materials		
(x) PPE (gloves, mask and glasses)	(x) Identification bracelet	( ) Ventilator
(x) Alcohol gel	(x) Patient's medical record	( ) Aspirator

Este manual operacional para o preenchimento do formulário de planejamento e elaboração de cenário, versão 1.2 de outubro de 2018 contém informações privilegiadas e/ou confidenciais de propriedade da **SIMSAFETY** Treinamento Desenvolvimento & Educação.
All use (economic or otherwise) depends on prior, formal and express authorization from the author, including partial or complete reproduction and use in any form. When using the document, it is mandatory to mention the name of the person who authored the work."
SIMSAFETY

(x) Stethoscope	( ) Specific forms	( ) Infusion pump
( ) Sphygmomanometer	( ) Intravenous infusions, specify:______	( ) Emergency vehicle
( ) Thermometer	( ) Medicines, specify:_______	( ) Defibrillator / cardioverter
( ) Tray	( ) Rigid plank	( ) BIPAP
( ) Material venous access	( ) Intubation material	( ) Scalpel
( ) Gauze	( ) Otoscope	( )
( ) Bandage	( ) Glucometer	( )
( )	( )	( )
( )	( )	( )

40.Ambiance/environment		
Scenario location:		
(x) Stretcher	(x) Telephone	( ) Armchair
(x) Serum support	( ) Computer	( ) Dining table
( ) Accessory table	( ) Cup	( ) Table
( ) Examination lamp	( ) Water	( ) Chair
( ) Step	( ) Tissue	( ) Others:
Ambient sound:	Configuration:	
( ) Ambulance siren	( ) continuous	( ) Interruption: specify time:___
( ) Background music	( ) continuous	( ) Interruption: specify time:___
( ) Beeping from monitors	( ) continuous	( ) Interruption: specify time:___
( ) Respiratory ventilator	( ) continuous	( ) Interruption: specify time:___
( ) Crying	( ) continuous	( ) Interruption: specify time:___
( ) Others:	( ) continuous	( ) Interruption: specify time:___

41. Make-up/*Moulage* and Accessories			
Location	Type	Size	Photo
Incision dressing	Bloody	Small amount of secretion	

PART VI: COMPLETE CASE DESCRIPTION

42. Scenario:	Chest drain

Este manual operacional para o preenchimento do formulário de planejamento e elaboração de cenário, versão 1.2 de outubro de 2018 contém informações privilegiadas e/ou confidenciais de propriedade da **SIMSAFETY** Treinamento Desenvolvimento & Educação.
All use (economic or otherwise) depends on prior, formal and express authorization from the author, including partial or complete reproduction and use in any form. When using the document, it is mandatory to mention the name of the person who authored the work."
SIMSAFETY

43.Case description: Pedro da Silva, 71 years old, admitted to the semi-intensive care unit on the 3rd postoperative day of lung adenocarcinoma removal.<br><br>Start of scenario:<br><br>Patient requests the presence of a professional from the nursing team.

Character History and Anamnesis			
44. Complete name:	Pedro da Silva		
45. Location:	Semi-intensive		
46.Medical record: 300800		47. Bed:1	
48. Weight: 60Kg	49. Height: 1.75cm	50. Allergies: negative	
51. Gender: ( ) M (x) F		52. Date of birth:01/05/1947	
53. Main complaint: 4 months of weight loss, dyspnea, persistent hoarseness and hemoptysis.			
54. History of current illness: He sought medical attention and was diagnosed with lung adenocarcinoma. He underwent lobectomy in the left hemithorax to remove the pulmonary adenocarcinoma..			
55. Family history: grandfather died of lung cancer.			
56. Social history: widowed, his granddaughter was born 2 months ago, so his daughter cannot follow his hospitalization full time. Works as a security guard in a condominium.			
57. Background: smoker, hypertensive, cholesterolemia, Acute Myocardial Infarction for 5 years.			
58. Medication use:<br>AAS® 100mg at lunch<br>Atenolol® 50 mg twice per day<br>Simvastatin® 20 mg once per day<br>Chlorthalidone® 12.5 mg twice per day			
59. Anamnesis	General		
	Dermatological	Cyanosis of extremities	
	Head and neck		
	Nervous		
	Respiratory	Dyspnea, decreased MV in bases, mainly on the right, snoring present	
	Cardiovascular		
	Gastrointestinal		
	Urinary		
	Genital		

Este manual operacional para o preenchimento do formulário de planejamento e elaboração de cenário, versão 1.2 de outubro de 2018 contém informações privilegiadas e/ou confidenciais de propriedade da **SIMSAFETY** Treinamento Desenvolvimento & Educação.
All use (economic or otherwise) depends on prior, formal and express authorization from the author, including partial or complete reproduction and use in any form. When using the document, it is mandatory to mention the name of the person who authored the work."
SIMSAFETY

	Skeletal muscle	
	Limbs/Peripheral Vascular	
	Psychological	
60.Previous exams performed	Collected exams in the morning routine	

Technical information		
61. Monitor: ( ) Off and not connected or<br>(x) Powered on and connected to:<br>62. (x) HR: 110<br>63. (x) Cardiac rhythm: sinus<br>64. (x) BP: 140/90<br>65. (x) RR: 32<br>66. (x) Temp.: 36.6<br>67. (x) Sat O_2: 89<br>68. ( ) CVP: ______<br>69: ( ) MAP: ______<br>70. ( ) Other parameters:___________		
71. CFT:	72. Capillary Glycemia:	73.Glasgow<br>(E V M)

Character Profile Description	
74.Psychological:	Oriented, aware, concerned about your current situation
75. Social:	Security of a condominium, with its source of income, helps your daughter, because her son-in-law is unemployed
76. Physical:	Simulator
77. Costume:	Nightdress
78. Technical:	Start scenario with horizontal decubitus
Scenario evolution	
79. Attitude A (Positive outcome)	81. Attitude B (Negative outcome)
If the volunteer(s) provide adequate communication and guidance, the patient will agree to the procedures and thank the staff.	If the volunteer(s) do not communicate properly, omitting information, the patient will be suspicious and insecure. Will maintain a questioning posture
80. Phrases which can be used	82. Phrases which can be used

Este manual operacional para o preenchimento do formulário de planejamento e elaboração de cenário, versão 1.2 de outubro de 2018 contém informações privilegiadas e/ou confidenciais de propriedade da **SIMSAFETY** Treinamento Desenvolvimento & Educação.
All use (economic or otherwise) depends on prior, formal and express authorization from the author, including partial or complete reproduction and use in any form. When using the document, it is mandatory to mention the name of the person who authored the work."
SIMSAFETY

“I don't know, the shortness of breath started after they changed the bottle, your colleagues from the previous shift”<br>“Something happened, why did my shortness of breath get worse?”<br>“Do you need to notify the doctor?”<br>“Will I have no problems? What can I do to help them”	“What do you mean your colleagues don’t know anything!"<br>“It has already happened to the patient next door; how can I trust this hospital!”<br>“I want to talk to my doctor now!”

83. Scenario evolution (Vital and clinical parameters)						
Condition	1 “declamping” the drain	2 Failure to identify the drain “clamping”	3	4	5	6*
Clinical	Improvement of the clinical picture	Worsening of the clinical picture				
Level of consciousness	aware and oriented	Conscious, complaining of severe shortness of breath				
Heart rate	sinus	sinus				
Cardiac rhythm	100	125				
BP	140/90	160/100				
RR	26	40				
Sat O_2	95	80				
Temperature	36.6	36.5				
$ETCO_2$						
Others:						
Actions and transitions	Evaluated and identified the drain “Clamping” within two minutes of beginning the scenario	Evaluated and performed several interventions such as elevation of the decubitus position and oxygen therapy, but did not identify the chest drain “clamping”				

Este manual operacional para o preenchimento do formulário de planejamento e elaboração de cenário, versão 1.2 de outubro de 2018 contém informações privilegiadas e/ou confidenciais de propriedade da **SIMSAFETY** Treinamento Desenvolvimento & Educação.
All use (economic or otherwise) depends on prior, formal and express authorization from the author, including partial or complete reproduction and use in any form. When using the document, it is mandatory to mention the name of the person who authored the work."
SIMSAFETY

* Add columns if necessary

PART VII: EXAM RESULTS

84. Laboratory or Reports			
Exams	Initial result	Final result	Normality values
Sodium			
Potassium			
Calcium			
Magnesium			
Hb	11		12.0 – 15.5 g/dL
Hematocrit	35		35 - 45%
Platelets			
Ultrasound			
Others:			

85. Images	

Este manual operacional para o preenchimento do formulário de planejamento e elaboração de cenário, versão 1.2 de outubro de 2018 contém informações privilegiadas e/ou confidenciais de propriedade da **SIMSAFETY** Treinamento Desenvolvimento & Educação.
All use (economic or otherwise) depends on prior, formal and express authorization from the author, including partial or complete reproduction and use in any form. When using the document, it is mandatory to mention the name of the person who authored the work."
SIMSAFETY

PART VIII: DEBRIEFING

86. Audio Video			
( ) Individual	(x) Group	(x) With video	( ) No video
87.Objective(s)	Identify the chest drain “clamping”;<br>Relate the signs and symptoms with possible complications of the patient with chest drain		
88.Suggested questions for the Debriefing	✓ Which interventions are effective?<br>✓ Did you feel comfortable in making a decision for the problems presented? If yes, why? If not, why?<br>✓ In your opinion, what did you identify as relevant?<br>✓ What did you learn in this scenario?<br>✓ Is it easy to approach the patient when a failure occurs? Should the patient be notified of the occurrence? If yes, why? if not, why?		
89.Critical points	CRM:<br>Call for help early<br>Communication<br>Use all information<br>Prevent and manage errors<br>Repeatedly re-evaluate<br>Set priorities<br>Others:<br>Identify the drain “clamping”<br>Evaluation<br>Ethics and professionalism		

PART IX: CHECK LIST/SKILLS PERFORMANCE ANALYSIS

90. Check List/Skills performance analysis		
**Scenario**	Chest drain	
**Topics**	**Actions**	**“X” for the performed actions**

Este manual operacional para o preenchimento do formulário de planejamento e elaboração de cenário, versão 1.2 de outubro de 2018 contém informações privilegiadas e/ou confidenciais de propriedade da **SIMSAFETY** Treinamento Desenvolvimento & Educação.
All use (economic or otherwise) depends on prior, formal and express authorization from the author, including partial or complete reproduction and use in any form. When using the document, it is mandatory to mention the name of the person who authored the work."

**Technical skills**	1. Initial assessment<br>2. Decubitus elevation<br>3. Monitoring vital signs<br>4. Installation of oxygen therapy<br>5. Identification of the drain “clamping”<br>6. “Unclamping”<br>7. Reassessment<br>8. Communicate medical staff	
**Patient and employee safety**	1. Patient identification<br>2. Hand hygiene<br>3. Locked of bed Rails<br>4. Use of PPE<br>5. Event Notification<br>6. Notes in the medical record	
**Non-technical skills**	1. Personal introduction<br>2.Patient guidance regarding the performed procedures<br>3. Ethics and professionalism<br>4. Prevent and manage errors<br>5. Reassessment of the framework repeatedly<br>6. Care prioritization<br>7. Communication<br>8. Situational awareness	

Additional comments:

PART X: CASE READING TO PARTICIPANT

91.Case reading – Scenario: Chest drain
**Unit: Semi-intensive**<br><br>**Professional: Nursing team**<br><br>**Case summary:**<br><br>Pedro da Silva, 69 years old, admitted to the semi-intensive care unit on the 3rd post-operative day of pulmonary adenocarcinoma removal.<br><br><u>Scenario start:</u>

Este manual operacional para o preenchimento do formulário de planejamento e elaboração de cenário, versão 1.2 de outubro de 2018 contém informações privilegiadas e/ou confidenciais de propriedade da **SIMSAFETY** Treinamento Desenvolvimento & Educação.
All use (economic or otherwise) depends on prior, formal and express authorization from the author, including partial or complete reproduction and use in any form. When using the document, it is mandatory to mention the name of the person who authored the work."
SIMSAFETY

Patient requests the presence of a professional from the nursing team.

Scenario agenda

Instructions: 5 minutes
Scenario: 10 minutes
Debriefing: 5 minutes

Este manual operacional para o preenchimento do formulário de planejamento e elaboração de cenário, versão 1.2 de outubro de 2018 contém informações privilegiadas e/ou confidenciais de propriedade da **SIMSAFETY** Treinamento Desenvolvimento & Educação.
All use (economic or otherwise) depends on prior, formal and express authorization from the author, including partial or complete reproduction and use in any form. When using the document, it is mandatory to mention the name of the person who authored the work."
SIMSAFETY

Annex 2 Example of a behavioral scenario

PART I: GENERAL PLANNING

64. Scenario:	Communicating bad news				
65. Institution of target audience:			xxxx		
66. Target audience:			Nurses		
67. Authors responsible for the construction:			Regina Mayumi Utiyama Kaneko<br>Fabiane Carvalhais Regis		
68. Construction date:		18/05/2016	69. Revision date:		
70. Pilot test date:		25/05/2016	71. Considerations regarding the pilot test:		
72. Instructor(s)/Facilitator(s):			Regina Mayumi Utiyama Kaneko<br>Fabiane Carvalhais Regis		
73. Scenario category, according to the number of specific objectives:			( ) A	( ) B	(x) C

PART II: MINIMUM REQUIREMENTS

74. Pre-simulation knowledge required:	Stages of the dying process<br>SPIKES Protocol
75. Pre-simulation activities required:	Dialogued expository class<br>Role Play<br>Discussion of videos
76. Suggested post-simulation activities:	Submitting an article for further reading
77. Pre-simulation evaluation:	Not performed
78. Post-simulation evaluation:	Not performed

PART III: LEARNING FROM RESULTS

79. General objective(s):	Sensitize nursing professionals to approach the patient and family in difficult situations
80. Specific objective(s):	Use communication strategies in difficult situations<br>Conduct the communication of bad news of a death of a child to the mother<br>Practice skills in communicating bad news using the SPIKES protocol
81. Relevant critical points of the scenario:	Personal presentation;<br>Certification of the child's mother;

Este manual operacional para o preenchimento do formulário de planejamento e elaboração de cenário, versão 1.2 de outubro de 2018 contém informações privilegiadas e/ou confidenciais de propriedade da **SIMSAFETY** Treinamento Desenvolvimento & Educação.
All use (economic or otherwise) depends on prior, formal and express authorization from the author, including partial or complete reproduction and use in any form. When using the document, it is mandatory to mention the name of the person who authored the work."
SIMSAFETY

( ) CRM (Crew Resource Management):<br><br>(x) Others:	Assertive communication (verbal and non-verbal)<br>Performing the steps of the SPIKES protocol
82. Expected results:	Participation rate above 90%<br>NPS (Net Promoter Score) above 80
83. Indicators:	Not applied
84. Opportunities to improve the scenario:	Volunteer felt lack of tissue to offer the actress at the time of bad news.<br>Guidance for the actress to maintain an initial profile of optimism with her daughter's condition.

PART IV: REFERENCES FOR THE PREPARATION OF CONTENT AND SUBMISSION OF THE MATERIAL FOR PRE-OR POST-SIMULATION READING

85. References
Maria Júlia Paes da Silva, "Comunicação de Más Notícias", O Mundo da Saúde, São Paulo - 2012;36(1):49-53
Como comunicar más notícias: revisão bibliográfica Rev. SBPH v.10 n.1 Rio de Janeiro jun. 2007

PART V: SIMULATION CENTER LOGISTICS

Volunteer information (Student/Participant)					
86. Volunteer(s):	Nurse				
87. Number of volunteers for the initial scenario:	(1)	(2)	(3)	(4)	(>4), specify: ______
88. Will the insertion of more volunteers be allowed after beginning the scenario?	(x) No	( ) Yes How many? - ________<br><br>Who?__________			

Duration	
89. Duration of instructions	5 minutes
90. Scenario duration time	10 minutes

Este manual operacional para o preenchimento do formulário de planejamento e elaboração de cenário, versão 1.2 de outubro de 2018 contém informações privilegiadas e/ou confidenciais de propriedade da **SIMSAFETY** Treinamento Desenvolvimento & Educação.
All use (economic or otherwise) depends on prior, formal and express authorization from the author, including partial or complete reproduction and use in any form. When using the document, it is mandatory to mention the name of the person who authored the work."
SIMSAFETY

91. Feedback of the standardized actor/actress/patient	5 minutes
92. Debriefing	20 minutes

Technological and Human Resources to compose the scenario character	
93. Simulator/Standardized patient	94. Name of character
( ) High-fidelity simulator	
( ) Medium-fidelity simulator	
( ) Low-fidelity simulator	
(x) Actor/actress	Carla Santos
( ) Hybrid	
( ) Instructor	
( ) Student/Participant	
( ) Patient	

General instructions				
Simulator/Standardized patient	32. Select the items to start the scenario	33. Wait outside the scenario	34. Describe the time to enter the scenario location	35. Describe the character's position in the setting for audio and video logistics
( ) High-fidelity simulator				
( ) Medium-fidelity simulator				
( ) Low-fidelity simulator				
(x) Actor/Actress	x			The actress must sit in the armchair on the left so that the zoom camera can focus on the volunteer
( ) Hybrid				
( ) Instructor				
( ) Student/Participant				
( ) Patient				
36. Keyword for scenario interruption:	Astronaut			

Este manual operacional para o preenchimento do formulário de planejamento e elaboração de cenário, versão 1.2 de outubro de 2018 contém informações privilegiadas e/ou confidenciais de propriedade da **SIMSAFETY** Treinamento Desenvolvimento & Educação.
All use (economic or otherwise) depends on prior, formal and express authorization from the author, including partial or complete reproduction and use in any form. When using the document, it is mandatory to mention the name of the person who authored the work."
SIMSAFETY

37. Monitoring	
( ) EkG	( ) ICP
( ) Oximetry	( ) Cardiac Output
( ) NIBP	( ) PAP
( ) IBP	( )
( ) CVP	( )
( ) Temperature	( )
( ) Capnography	( )

38. Acesses	
Catheters / Drains / Dressings	Location and characteristic
( ) Peripheral Venous Access:	
( ) Central Venous Access:	
( ) Intubation:	
( ) Oxygen therapy:	
( ) NG tube:	
( ) NE tube:	
( ) Urinary catheter:	
( ) Chest drain:	
( ) JP drain:	
( ) Incisions:	
( ) Dressings:	
( )	
( )	

39.Equipment and Materials		
( ) PPE (glove, mask and glasses)	( ) Identification bracelet	( ) Ventilator
( ) Alcohol gel	( ) Patient's medical record	( ) Aspirator
( ) Stethoscope	( ) Specific forms	( ) Infusion pump
( ) Sphygmomanometer	( ) Intravenous infusions, specify:_______	( ) Emergency vehicle
( ) Thermometer	( ) Medicines, Specify:________	( ) Defibrillator / cardioverter
( ) Tray	( ) Rigid plank	( ) BIPAP
( ) Material venous access	( ) Intubation Material	( ) Scalpel
( ) Gauze	( ) Otoscope	( )

Este manual operacional para o preenchimento do formulário de planejamento e elaboração de cenário, versão 1.2 de outubro de 2018 contém informações privilegiadas e/ou confidenciais de propriedade da **SIMSAFETY** Treinamento Desenvolvimento & Educação.
All use (economic or otherwise) depends on prior, formal and express authorization from the author, including partial or complete reproduction and use in any form. When using the document, it is mandatory to mention the name of the person who authored the work."
SIMSAFETY

( ) Bandage	( ) Glucometer	( )
( )	( )	( )
( )	( )	( )

40. Ambiance / Environment		
Scenario location:		
( ) Stretcher	( ) Telephone	(x) Armchair - 2
( ) Serum support	( ) Computer	( ) Dining table
( ) Accessory table	(x) Cups	( ) Table
( ) Examination Lamp	(x) Water	( ) Chair
( ) Step	(x) Tissue	(x) Others: Bag with belongings (12-year-olds' clothes)
Ambient sound:	Configuration:	
( ) Ambulance siren	( ) continuous	( ) Interruption: specify time:___
( ) Background music	( ) continuous	( ) Interruption: specify time:___
( ) Beeping from monitors	( ) continuous	( ) Interruption: specify time:___
( ) Respiratory ventilator	( ) continuous	( ) Interruption: specify time:___
( ) Crying	( ) continuous	( ) Interruption: specify time: ____
( ) Others:	( ) continuous	( ) Interruption: specify time:___

41. Make-up/Moulage and Accessories			
Location	Type	Size	Photo
Face	Pallor/pastiness	Accentuated	

PART VI: COMPLETE CASE DESCRIPTION

42.Scenario:	Communicating bad news
43.Case description:	

All use (economic or otherwise) depends on prior, formal and express authorization from the author, including partial or complete reproduction and use in any form. When using the document, it is mandatory to mention the name of the person who authored the work."

Clara Santos, 12 years old, was hospitalized in the Intensive Care Unit for 3 days. A patient with ALL (Acute Lymphoblastic Leukemia) presented pulmonary infection, followed by septic shock, was intubated, was on mechanical ventilation and sedated. She had cardiorespiratory arrest and was unsuccessfully resuscitated. Death was found by the on-call doctor and he reported the death to Clara's mother.<br><br>Start of scenario:<br><br>You are a nurse who starts your work shift and received the shift with your colleague. Clara's mother is in the room attached to the ICU and you need to deliver Clara's belongings to the mother.

Character History and Anamnesis		
44.Complete name:	Clara dos Santos	
45. Location:	Pediatric ICU	
46. Medical record: 322240		47. Bed:2
48. Weight:42	49. Height: 1.62	50. Allergies: negative
51.Gender: ( ) M (x) F		52.Date of birth: 05/06/2006
53. Main complaint: Complaints: shortness of breath, fever, weakness, pallor, generalized body pain.		
54. History of current illness: for 4 months, weakness, pallor, fever, bone pain and the appearance of bruises on the body. She was diagnosed with ALL and started chemotherapy treatment 3 months ago. The response to treatment is not as satisfactory as the medical team would like, and bone marrow transplantation has been discussed in the last chemo session.		
55. Family history:		
56. Social history: Clara has a 9-year-old sister Maria. Ana, the mother is an architect and in her current condition, she passed all the work to the company partner. Ana's father is an engineer. The whole family has been taking turns to stay with Clara in the hospital for hospitalizations and chemotherapy sessions.		
57. Family background:		
58. Medication use:		
59. Anamnesis	General	
	Dermatological	
	Head and neck	
	Nervous	
	Respiratory	
	Cardiovascular	
	Gastrointestinal	
	Urinary	
	Genital	

Este manual operacional para o preenchimento do formulário de planejamento e elaboração de cenário, versão 1.2 de outubro de 2018 contém informações privilegiadas e/ou confidenciais de propriedade da **SIMSAFETY** Treinamento Desenvolvimento & Educação.
All use (economic or otherwise) depends on prior, formal and express authorization from the author, including partial or complete reproduction and use in any form. When using the document, it is mandatory to mention the name of the person who authored the work."

	Skeletal muscle	
	Limbs/Peripheral Vascular	
	Psychological	
60. Previous exams performed		

Technical information
61. Monitor: ( ) Off and not connected or<br>( ) Powered on and connected to:<br>62. ( ) HR: _______<br>63. ( ) Cardiac rhythm: _______<br>64. ( ) BP: _______<br>65. ( ) RR: _______<br>66. ( ) Temp.: _______<br>67. ( ) Sat O_2: _______<br>68. ( ) CVP: _______<br>69: ( ) MAP: _______<br>70. ( ) Other parameters:___________

71.CFT:	72. Capillary Glycemia:	73.Glasgow<br>(E V M)

Character Profile Description: Carla Santos	
74.Psychological:	Sensitive, exemplary mother, does everything to support her daughter's fight against this cancer, is not discouraged by anything, waits in the room next to the ICU, as the nursing team asked her to leave for the procedure. The doctor on duty talked to Ana and reported Clara's death. However, Carla did not understand and in her view, her daughter is in the ICU in serious condition and recovering. She will initially be confused and anxious for information.
75.Social:	Architect. She encourages her daughters as they both enjoy arts.
76.Physical:	The actress' physique

Este manual operacional para o preenchimento do formulário de planejamento e elaboração de cenário, versão 1.2 de outubro de 2018 contém informações privilegiadas e/ou confidenciais de propriedade da **SIMSAFETY** Treinamento Desenvolvimento & Educação.
All use (economic or otherwise) depends on prior, formal and express authorization from the author, including partial or complete reproduction and use in any form. When using the document, it is mandatory to mention the name of the person who authored the work."
SIMSAFETY

77.Costume:	Casual clothes	
78.Technical:	SPIKES Protocol	

Scenario evolution	
79. Attitude A (Positive outcome)	81. Attitude B (Negative outcome)
If the volunteer demonstrates welcoming and empathetic behavior, explain Clara's condition if presented (name, position and appropriate body posture); build rapport and empathy, welcoming Ana who shows she doesn't understand the situation; assertive communication (congruence between what he says and what he expresses, verbal and non-verbal); obtain and transmit necessary information; listen actively, the actress must accept the reception at the end of the scenario	If the volunteer delivers the belongings and is indifferent to Ana's emotional state, she will present a reactive posture, denial and will question some facts as described below…
80. Phrases which can be used	82. Phrases which can be used
"Hi! Is Clara better? Did you manage to perform the procedure? I was asked to leave her side for a while."<br>"Her condition is serious, but she has been through so much worse. You know, she is a warrior, a wonderful daughter, always with a smile on her face despite the pain."<br>"This morning, despite the sedation, I held her little hand and talked to her, we remembered the family moments and the funny things she did! She has often made us strong. It should be the other way around, shouldn't it? The nurse said it was okay for me to talk to her and touch her."<br>"The Doctor came here to talk to me and said that her condition is serious, I didn't understand very well, she spoke with many terms that you use. I can go into the ICU now and be by her side!"<br>"It's not possible, her heart was beating normally, I know her condition is serious, but what do you mean, she died! It's not possible! It can't be!"<br>"My husband has just left the ICU, he spent the night with her!"	"How can that be?!! When I left her side, it was all right!"<br>"What did you do?!!"<br>"Her condition is serious, but everything was going well, it was just the changing shifts when this happened!!"<br>"I want to talk to the doctor, I don't want to talk to you"<br>"Do you have kids?!!"<br>"Can you imagine my pain??"<br>"Are you sure they did everything they could?!!"<br>"You are not telling the truth!"<br>"I want to enter the ICU now, who is going to stop me, that security guard over there???"<br>"You didn't even look me in the eye, you come here and you tell me this with this indifference!"<br>"How is this possible??"<br>"I can't believe it, it can't be true!"<br>"You guys did something wrong, I'm going to sue this hospital!"

Este manual operacional para o preenchimento do formulário de planejamento e elaboração de cenário, versão 1.2 de outubro de 2018 contém informações privilegiadas e/ou confidenciais de propriedade da **SIMSAFETY** Treinamento Desenvolvimento & Educação.
All use (economic or otherwise) depends on prior, formal and express authorization from the author, including partial or complete reproduction and use in any form. When using the document, it is mandatory to mention the name of the person who authored the work."
SIMSAFETY

"Please say it’s not true!"<br>-crying-<br>“I have another daughter, Maria. She cut her hair and gave it to Clara to make a wig. What am I going to tell her??!<br>“Is it her clothes? – Open the bag and take the drawing – “it’s the drawing she made when she was in the room.<br>-crying and silence - (don't speak)<br>"I'll never be able to hug her again, make plans, there’s an emptiness as if a part were ripped off!!"<br>“Even knowing that one day life ends, we are not prepared to lose someone we love so much!	- can get out of control and increase the level of anxiety -

83.Scenario evolution (Vital and Clinical Parameters)						
Condition	1	2	3	4	5	6*
Clinical						
Level of consciousness						
Heart rate						
Cardiac rhythm						
BP						
RR						
Sat O_2						
Temperature						
$ETCO_2$						
Others:						
Actions and transitions						

Este manual operacional para o preenchimento do formulário de planejamento e elaboração de cenário, versão 1.2 de outubro de 2018 contém informações privilegiadas e/ou confidenciais de propriedade da **SIMSAFETY** Treinamento Desenvolvimento & Educação.
All use (economic or otherwise) depends on prior, formal and express authorization from the author, including partial or complete reproduction and use in any form. When using the document, it is mandatory to mention the name of the person who authored the work."
SIMSAFETY

* Add columns if necessary

PART VII: EXAM RESULTS

84. Laboratory or Reports			
Exams	Initial result	Final result	Normality values
Sodium			
Potassium			
Calcium			
Magnesium			
Hb			
Hematocrit			
Platelets			
Ultrasound			
Others:			

85.Images	

PART VIII: DEBRIEFING

86.Audio Video			
(x) Individual	( ) Group	(x) With video	( ) No video

87.Objective(s)	Use communication strategies in difficult situations;<br>Conduct the communication of bad news of a death of a child to the mother;<br>Practice skills in communicating bad news using the SPIKES protocol.
88. Suggested questions for the Debriefing	➢ Did you feel uncomfortable for this bad news communication? If yes, why?<br>➢ In your opinion, what was the most important communication during the scenario?<br>➢ What behaviors and attitudes were effective, and why?<br>➢ Would you do it differently next time?<br>➢ What did you learn in this scenario??"
89.Critical points	➢ Personal presentation;<br>➢ Certification of the child's mother;<br>➢ Assertive communication (verbal and non-verbal)<br>➢ Performing the steps of the SPIKES protocol

Este manual operacional para o preenchimento do formulário de planejamento e elaboração de cenário, versão 1.2 de outubro de 2018 contém informações privilegiadas e/ou confidenciais de propriedade da **SIMSAFETY** Treinamento Desenvolvimento & Educação.
All use (economic or otherwise) depends on prior, formal and express authorization from the author, including partial or complete reproduction and use in any form. When using the document, it is mandatory to mention the name of the person who authored the work."
SIMSAFETY

PART IX: CHECK LIST/SKILLS PERFORMANCE ANALYSIS

90.Check List/Skills performance analysis		
**Scenario**	Communicating bad news	
**Topics**	**Actions**	**"X" for the performed actions**
**Non-technical skills**	1. S (Review the data, assess your own feelings about the delivery of this bad news, environment, plan the time, involve people)<br>2. P (Rapport, active listening, establishing initial dialogue, allowing questions, considering personal aspects, avoiding judgment)<br>3. I (Investigate the need for detailed information, offer yourself and answer any question, have the sensitivity to see if you are in a position to receive the news)<br>4. K (Inform clearly and sincerely, avoid technical terms, professionalism)<br>5. E (Favoring the expression of the family member, welcoming, enduring the discomfort of the situation)<br>6. S (Summarize key issues addressed, share decisions made, and be honest)	

Additional comments:

Este manual operacional para o preenchimento do formulário de planejamento e elaboração de cenário, versão 1.2 de outubro de 2018 contém informações privilegiadas e/ou confidenciais de propriedade da **SIMSAFETY** Treinamento Desenvolvimento & Educação.
All use (economic or otherwise) depends on prior, formal and express authorization from the author, including partial or complete reproduction and use in any form. When using the document, it is mandatory to mention the name of the person who authored the work."
SIMSAFETY

PART X: CASE READING TO PARTICIPANT

91.Case reading – Scenario: Communication of bad news
**Unit:** Intensive Care Unit waiting room<br><br>**Professional:** Nurse<br><br>**Case summary:**<br><br>Clara Santos, 12 years old, was hospitalized in the Intensive Care Unit for 3 days. A patient with ALL (Acute Lymphoblastic Leukemia) presented with pulmonary infection, followed by septic shock, was intubated, was on mechanical ventilation and sedated. She had cardiorespiratory arrest and was unsuccessfully resuscitated. Death was found by the on-call doctor and he reported the death to Clara's mother<br><br><u>Scenario start:</u><br><br>You are a nurse who starts your work shift and received the shift with your colleague. Clara's mother is in the room attached to the ICU and you need to deliver Clara's belongings to the mother.<br><br>**Scenario agenda**<br><br>Instructions: 5 minutes<br>Scenario: 10 minutes<br>Debriefing/Feedback from the actress: 5 minutes

Este manual operacional para o preenchimento do formulário de planejamento e elaboração de cenário, versão 1.2 de outubro de 2018 contém informações privilegiadas e/ou confidenciais de propriedade da **SIMSAFETY** Treinamento Desenvolvimento & Educação.
All use (economic or otherwise) depends on prior, formal and express authorization from the author, including partial or complete reproduction and use in any form. When using the document, it is mandatory to mention the name of the person who authored the work."
SIMSAFETY

REFERENCES

Issenberg SB, McGaghie WC, Petrusa ER, Lee Gordon D, Scalese RJ. Features and uses of high-fidelity medical simulations that lead to effective learning: a BEME systematic review. Med Teach. 2005;27(1):10-28

INACSL Standards of best practice: Simulation SM Simulation design. Clinical Simulation in Nursing.2016;(12):5-12.

Jeffries PR. A framework for designing, implementing, and evaluating simulations used as teaching strategies in nursing. Nurs Educ Perspect 2005;26(2):96-103.

Motola I, Devine LA, Chung HS, Sullivan JE, Issenberg SB. Simulation in healthcare education: a best evidence practical guide. AMEE Guide No. 82. Med Teach. 2013;35(10):e1511-30. DOI: 10.3109/0142159X.2013.818632

Este manual operacional para o preenchimento do formulário de planejamento e elaboração de cenário, versão 1.2 de outubro de 2018 contém informações privilegiadas e/ou confidenciais de propriedade da **SIMSAFETY** Treinamento Desenvolvimento & Educação.
All use (economic or otherwise) depends on prior, formal and express authorization from the author, including partial or complete reproduction and use in any form. When using the document, it is mandatory to mention the name of the person who authored the work."
SIMSAFETY
